# Integrative Study of Physiological Changes Associated with Bacterial Infection in Pacific Oyster Larvae

**DOI:** 10.1371/journal.pone.0064534

**Published:** 2013-05-21

**Authors:** Bertrand Genard, Philippe Miner, Jean-Louis Nicolas, Dario Moraga, Pierre Boudry, Fabrice Pernet, Réjean Tremblay

**Affiliations:** 1 Institut des sciences de la mer, Université du Québec à Rimouski, Rimouski, Québec, Canada; 2 Unité de recherche Physiologie Fonctionnelle des Organismes Marins, Laboratoire des Sciences de l′Environnement Marin (LEMAR), Institut Français de Recherche pour l′Exploitation de la Mer (IFREMER), Plouzané, France; 3 Laboratoire des Sciences de l′Environnement Marin (LEMAR), Institut Universitaire Européen de la Mer, Université de Bretagne Occidentale, Plouzané, France; Rush University Medical Center, United States of America

## Abstract

**Background:**

Bacterial infections are common in bivalve larvae and can lead to significant mortality, notably in hatcheries. Numerous studies have identified the pathogenic bacteria involved in such mortalities, but physiological changes associated with pathogen exposure at larval stage are still poorly understood. In the present study, we used an integrative approach including physiological, enzymatic, biochemical, and molecular analyses to investigate changes in energy metabolism, lipid remodelling, cellular stress, and immune status of *Crassostrea gigas* larvae subjected to experimental infection with the pathogenic bacteria *Vibrio coralliilyticus*.

**Findings:**

Our results showed that *V. coralliilyticus* exposure induced (1) limited but significant increase of larvae mortality compared with controls, (2) declined feeding activity, which resulted in energy status changes (i.e. reserve consumption, β-oxidation, decline of metabolic rate), (3) fatty acid remodeling of polar lipids (changes in phosphatidylinositol and lysophosphatidylcholine composition`, non-methylene–interrupted fatty acids accumulation, lower content of major C_20_ polyunsaturated fatty acids as well as activation of desaturases, phospholipase and lipoxygenase), (4) activation of antioxidant defenses (catalase, superoxide dismutase, peroxiredoxin) and cytoprotective processes (heat shock protein 70, pernin), and (5) activation of the immune response (non-self recognition, NF-κκ signaling pathway, haematopoiesis, eiconosoids and lysophosphatidyl acid synthesis, inhibitor of metalloproteinase and antimicrobial peptides).

**Conclusion:**

Overall, our results allowed us to propose an integrative view of changes induced by a bacterial infection in Pacific oyster larvae, opening new perspectives on the response of marine bivalve larvae to infections.

## Introduction

Bacterial infections can have serious consequences on the survival of bivalve larvae depending on host–pathogen interactions. When a pathogen infects a host, multiple reactions occur, initiated both by the pathogen in an attempt to survive and multiply, and by the host in an attempt to eliminate the pathogen. For the host, changes induced by infection can be seen at several levels such as molecular, physiological and biochemical processes. First, infection induces the activation of both cellular and humoral immune responses that act together to kill and eliminate the infecting bacteria. In bivalves, immunity is constituted of innate processes including various serologically active molecules (opsonins, lysins, agglutinins, and antimicrobial factors) and of the phagocytosis accompanied by production of oxygen metabolites and the release of lysosomal enzymes [1], [2]. During the last decade, knowledge of immune processes in adult bivalves has been significantly improved by the development of genomic tools [3], [4], [5].However, the immune characteristics of larvae remain underinvestigated, notably due to the difficulty to isolate haemocytes from larvae. Elston and Leibovitz [6] observed phagocytes (described as coelomocytes) containing bacterial fragments in the visceral cavity of *Crassostrea virginica* veliger larvae. Recently, the work of Tirape *et al.* [7] suggested that haemocytes appear during the gastrula–trochophore developmental stages. In the blue mussel *Mytilus galloprovincialis*, the antimicrobial peptides mytilin and defensin have been found during and after larval metamorphosis [8]. Furthermore, lysozyme-like and hydrolytic enzyme activities are present in *C. gigas* larvae and juveniles [9], [10]. Genomics approaches of immune-related genes expression in oyster larvae suggested that immune processes like Rel/NF-κB pathway, metalloproteinase inhibitor, non-self recognition, apoptosis and inflammatory response regulation occur as soon as larval stage [7], [11]. Bivalve haemocytes seem to respond to bacterial stimulation with a burst of respiratory activity similarly to the respiratory burst of mammalian phagocytes, resulting in the generation of various free radicals or reactive oxygen species (ROS) that eliminate the phagocytized material [12], [13]. ROS production are controlled by antioxidant defense systems to limit tissue peroxidation. Several studies have highlighted the importance of these antioxidant defenses during summer mortality in *C. gigas* [3], [14] and during mortality events in *C. virginica* larvae [15].

Pathogen exposure can also induce significant changes in larval energy metabolism. In *C. virginica* larvae, emergence of massive mortality coincided with both a decline in feeding activity and a metabolic depression, which could reduce the available energy for the immune response [15]. The energetic status of larvae can be estimated by lipid content, mainly triacylglycerols (TAG), which constitutes the main energy source during larval development [16], [17]. Previous studies showed that TAG levels were higher in *P. magellanicus* sea scallops larvae treated with an antibiotic than in untreated controls showing significant mortality [18]. Similar results were found recently in C. *virginica* larvae when massive mortality happened [15].

Beside their energetic function, lipids (especially polyunsaturated fatty acids: PUFA) are known to be essential for the development of bivalve larvae, especially to sustain growth and improve survival [19], [20]. Moreover, C_20_ PUFAs, such as eicosapentaenoic acid (EPA, 20∶5[n-3]) and arachidonic acid (AA, 20∶4[n-6]), are precursors of eicosanoids, a group of highly biologically active hormones including prostaglandins, leukotrienes, and hydroxyeicosatetraenoic acids [21]. Eicosanoid production is associated with stressful conditions. For example, excess eicosanoid production has been recorded under pathological conditions in invertebrates, especially insects [22]. AA levels in *P. magellanicus* showed a marked increase during the metamorphic stage co-occurring with elevated mortality and the establishment of pathogenic and opportunistic microbes in larval cultures [23]. Bacterial pathogens could also affect directly the composition of structural lipids and induce the degradation of tissue membranes by extracellular enzymes like phospholipases [24], [25].

In the present study, all these aspects were investigated using physiological, enzymatic, biochemical and molecular analysis in order to propose an integrative view of changes associated to bacterial infection in *C. gigas* larvae. Briefly, we used a bacterial challenge with *Vibrio coralliilyticus* on *C. gigas* larvae to evaluate the impact of pathogen exposure (1) on larval survival, growth, and feeding activity, (2) on energetic metabolism activity, (3) on the content of protein, carbohydrate, lipid classes, and fatty acids in larvae, (4) on antioxidant defense activity and lipid peroxidation, and (5) on the expression levels of candidate genes involved in energy metabolism, lipid metabolism, cellular stress and immunity.

## Methods

### Rearing procedures and experimental design

This project was conducted at the Ifremer facilities in Plouzané (Brittany, France) and took place from mid-May to June 2008. Twenty adult oysters *C. gigas* were conditioned at the Ifremer experimental station in Argenton (Brittany, France). The spawning was induced by thermal shock and the fertilized eggs were transferred to a 150 L cylindro-conical tank. Larvae (20 ind. mL^−1^ initially) were reared in the same kind of tank at 20°C in UV-treated filtered seawater with gentle aeration. Larvae were fed a mixture of *Isochrysis affinis galbana* and *Chaetoceros calcitrans* and food supply was carried out at each two days when water was renewed. Twelve days after fertilization (dpf), larvae were collected on a 100 µm square mesh filter, visually counted, pooled, and distributed equally into 10 L beakers (∼200×10^3^ larvae per beaker). Larvae were cultured in duplicate with or without pathogen challenge (10^4^ bacteria per mL in final concentration) over a period of 24 h or 48 h for a total of eight beakers (two unchallenged and challenged batches larvae per sampling time). Larvae were fed at the beginning of the experiment and samples were collected for physiological, biochemical, and molecular analyses at the end of the experiment at 24 h or 48 h (pool of ∼40×10^3^ larvae per sample for each kind of analysis). An additional sample of larvae (12 dpf) was also collected before pathogen inoculation to be used as reference sample. Seawater from carboys was sampled for measurements of particles to estimate feeding activity and for microbiological analysis. Samples were immediately flash frozen in liquid nitrogen and stored at –80°C. The pathogen used for bacterial challenge was *V. coralliilyticus* strain LPI 06/210 (16S rRNA accession number: HF549288) isolated from diseased oyster larvae. Although this bacterium was first described as a pathogen of coral [26], recent experimental infection demonstrated that this strain was strongly virulent to oyster larvae and provoked mass mortalities in oyster hatchery [27]. Prior to use this *V. coralliilyticus,* the strain kept at –80°C was streaked onto marine agar gel to ensure purity and was further cultured in marine broth (Difco, Lawrence, KS, USA) and incubated at 25°C 24 h prior to use. Bacterial culture was centrifuged (6000 g, 15 min), the supernatant was discarded and the pellet was resuspended in 10 mL of sterile seawater. Optical density of bacterial suspension was read at 600 nm and compared to a standard of sterile seawater to determine the bacterial concentration. Correspondence between optical density and bacterial counts on marine agar was previously performed in the lab.

### Larval performance

Shell length (SL), survival rate (% of live larvae), and feeding activity (number of cleared algal cells per larva per day) were estimated for each sample. Alive and dead larvae were distinguished and counted visually using a binocular microscope (Leitz Biomed, Wetzlar, Germany) in samples of 100–200 individuals. SL was measured with an inverted microscope (Leitz Leica DMIL, Wetzlar, Germany) equipped with a digital camera. Images were captured using the Turbo TV software and analyzed by Image SXM. Microalgal cell concentration was measured in triplicate with a Beckman-Coulter Z2 electronic particle counter (Beckman, USA) before and after experiments. Daily feeding activity was estimated as clearance rate = ((N_t = 0_-N_t = final_)/number of larvae per mL)/ number of days, where N is the concentration of microalgal cells per mL introduced (t = 0) and at the end of experiment (t = final).

### Lipid class and fatty acid analyses

Methods used to determine profiles of lipid classes and fatty acids were described in Haberkorn *et al*. [28] and Pernet *et al*. [29] respectively. Briefly, after extraction in a CHCl_3_–CH_3_OH mixture, neutral and polar lipids from samples of larvae and microalgae were extracted using silica gel micro-columns. Neutral and polar fractions were used for lipid classes and fatty acid determination. For lipid class determination, samples were spotted on activated silica plates using a CAMAG automatic sampler (CAMAG, Switzerland) and plates were eluted in specific solvent to separate neutral and polar classes. Lipid classes appeared as black spots after plates were dipped in a CuSO_4_–H_3_PO_4_ solution and heated. Plates were read by scanning at 370 nm, and black spots were quantified using Wincats software (CAMAG, Switzerland). Five neutral lipid classes (free fatty acids [FFA], alcohols [AL], mono-diacylglycerols [DG-MG], triacylglycerols [TAG], sterols [ST]) and seven polar lipid classes (cardiolipin or bisphosphatidylglycerol [Cardio], lysophosphatidylcholine [LPC], phosphatidylcholine [PC], phosphatidylethanolamine [PE], phosphatidylserine [PS], phosphatidylinositol [PI], ceramide aminoethylphosphonate [CAEP]) were identified based upon authentic standards (Sigma–Aldrich, St Louis, MO, USA). Results were expressed as a percent proportion of the mass of total lipids. Masses of neutral and polar lipids were assessed by summation of lipid classes and expressed by number of larvae. Analyses of fatty acid profiles were also performed on neutral and polar lipids. The transesterified lipids (FAME) were analyzed by gas chromatograph (Hewlett Packard, Palo Alto, CA, USA) using a DB-Wax (30 m×0.25 mm; 0.25 µm film thickness) capillary column coupled with a flame ionization detector. Fatty acids were identified by comparing their retention times with those of standards using the Chem Station software (Hewlett Packard, Palo Alto, CA, USA). Here again, results for fatty acid profiles were expressed as a percentage of the total fatty acid mass of each lipid fraction. The peroxidation index (PInd) was calculated as PInd = 0.025 (% monoenoics)+1×(% dienoics)+2×(% trienoics)+4×(% tetraenoics)+6×(% pentaenoics)+8×(% hexaenoics).

### Spectrophotometric analyses

Samples for spectrophotometric analysis were homogenized on ice in phosphate buffered saline (PBS: 80 mM; pH 7.6 at 25°C) with 0.1% (v/v) triton X-100 using a polytron homogenizer. A fraction of the homogenate was stored at –80°C for the determination of total carbohydrate and protein concentrations. The remaining sample was centrifuged (15,000 g) at 4°C for 10 min and the supernatant collected and stored at –80°C. Total carbohydrate and protein contents were measured colorimetrically as described by DuBois *et al.* [30] and Lowry *et al.* [31] respectively. The activities of seven enzymes were recorded in this study. Three were related to energy metabolism (pyruvate kinase, citrate synthase, cytochrome c oxidase) and four to antioxidant defenses (catalase, glutathione peroxidase, glutathione reductase, superoxide dismutase). The oxidative stress level was evaluated by measuring the lipid peroxidation. Methods for assaying enzyme activities of citrate synthase (CS), cytochrome c oxidase (CCO), catalase (CAT), glutathione peroxidase (GPX), superoxide dismutase (SOD), and the determination of lipid peroxidation levels (TBARS) were described in Genard *et al.* [15]. Pyruvate kinase (PK) activity was assayed according to the protocol of Childress and Somero [32] adapted for microplate reader [33] and the glutathione reductase (GR) activity was determined according to Smith *et al.* [34] after modification for microplate reader.

### RNA extraction and real-time PCR

Total RNA was extracted for each larva sample using TRIzol™Reagent (Invitrogen, Carlsbad, CA, USA) according to the manufacturer's protocol. RNA was resuspended in RNase-free water and concentrations were determined using a Nanodrop spectrophotometer (Thermofisher, Wilmington, DE, USA). RNA extracted from larvae was reverse transcribed using the RevertAid H Minus First Strand cDNA synthesis kit (Fermentas, Burlington, ON, Canada). Real-time PCR was performed on cDNA (1/120 dilution) using a 7300 Real-Time PCR System (Applied Biosystems, Foster City, CA, USA). Reaction component concentrations and real-time PCR settings were described in Genard *et al.* [11]. Each run included a negative control (non-reverse transcribed total RNA) and blank controls (water) for each primer pair. The threshold value (Ct) was determined for each gene as the number of cycles at which the fluorescence curve entered exponential phase. A sequential dilution of cDNA was performed for each set of primers in order to estimate the amplification efficiency using the equation Efficiency = 10^(−1/slope)^−1. The relative quantification (RQ) of each gene expression was calculated according to the comparative Ct method [35] using the formula RQ = (E_TG_)^(Ct HG(cal) - Ct HG(spl))^/ (E_HG_)^(Ct TG(cal) - Ct TG (spl))^, where E_TG_ and E_HG_ are the amplification efficiencies of the target and housekeeping (ribosomal 18S) genes, respectively, and Ct HG and Ct TG are the threshold values of the housekeeping and target genes in the calibrator (cal) or target sample (spl). Larvae sampled before pathogen challenge were used for calibration. Oligonucleotide primer sequences used to amplify specific gene products are given in Table 1. Selected genes were classified into four groups: energy metabolism, lipid metabolism, cellular stress and immunity.

**Table 1:** Details of genes and primers used in the quantitative real-time PCR relative expression analysis.

Genes	Label	GENBANK	Function	Forward primer (5′-3′)	Reverse primer (5′-3′)
a-agglutinin attachment subunit	AGL	CU984122	Non-self recognition (lectin family)	GCCTCCTTCTACACCACAGCAT	TGGTTTGCTTGGATTTACAGACTTC
Annexin 6	ANX6	CU989663	Apoptose, inflammation response	GTGACGATGATGCGGATGAG	GATTTTTCGCCAGACGATTACAC
c-type lectin-1	cLEC	CU992287	Non-self recognition (lectin family)	ACCTGGTCCGACGCAAGA	GGGTGCTCAGAAACTTGTTGATG
Cg-defensin 2	Defh2	AJ565499	Antimicrobial peptide	GTATTCGTACTTCTTACATTAGC	GCTCTACAACCGATGGACCT
Cg-DRAC3	DRAC3	BQ427023	Haemocytes proliferation	GATAATAGTGCGACGGAGTG	CATCAGCATACAGGTCTTCC
ECSIT	ECSIT	BQ427193	Immune response signalling (NF-KB)	CCTCATCGGGAATCACACCATTA	CGTGCGAGGGCATATAGAG1TTG
Galectin 8	Gal8	BQ427054	Non-self recognition (lectin family)	TGGAAGTTGAATCTGGTCTGG	TGCTGTTAAGAACCATCTCACG
Cg-LBI/BPI (LPS binding/bactericidal-permeability-increasing protein)	LBP/BPI	AY165040	Non-self recognition (LPS binding protein)	TGTCCTTGGCGACGGTCAGTTGTG	GTCCTCAAATTACCTATATCAGTAAC
Mitogen activated protein kinase kinase 1	MAPK	CU996721	Immune response signaling	AAACTGGCAAACCCCTGAAA	CCAGGCGGACCAGGAAA
Cg-MyD88 (Myeloid differentiation primary response (88))	MYD88	DQ530619	Immune response signaling (NF-KB)	AGGTACCGGCTGTGATACGA	TTCAAACGCCACCAAGACTG
Cg-REL	REL	AY039648	Immune response signaling (NF-KB)	GCTACGAGTGTGAGGGGAGATCA	GGGAAACTGATGACGTTGGTGTC
Cg-TAL (hematopoietic transcription factors)	TAL	AY039650	Haemocyte proliferation	CGTGTTGTGTCCGAGTGTATGTAA	GCTGTCGTCGCATTCTTTCA
Cg-TIMP (tissue inhibitor metalloproteinase)	TIMP	AF321279	Metalloproteinase inhibitor	CAGGGTCTTACAACACGAACGA	GCTGGTTTGGTTCACGGTAGA
cg-TRAF (TNF receptor associated factor)	TRAF	BQ426746	Immune response signaling (NF-KB)	CAGCCAGCCATTTTACACCAGTC	CCGTTTAAAACTGCTGCTTGTGG
Arachidonate 15-lipoxygenase	AA15LX	CU998478	Eicosanoide synthesis	CTCACTGCCCGCTTTCCA	GAGCGAGGAAGCGGAAGAG
Acyl-CoA synthetase	ACS	CU992135	Fatty acid oxidation	TACTGTCTTCTGCTAAACGCCAC	GATCATGTTTGTTCGGTCATC
Acyl-CoA dehydrogenase	ADH	FP001142	Fatty acid oxidation	CCGCTCACAATTCCACACAA	GCACCCCAGGCTTTACACTTT
Stearoyl-desaturase 5	Delta 5	CU997931	Fatty acid desaturation	GGAGGACTCTGAGCCCGAAT	GGCGAGGTGAAGGGAAGAC
Fatty acid desaturase 2	Delta 6	CU994528	Fatty acid desaturation	GAACTTTCGCCATTTTCAGCAT	GGCTATATCGACATCAGGGTCTTT
Delta 9 desaturase	Delta 9	CX069227	Fatty acid desaturation	TACTGTCTTCTGCTAAACGCCAC	GTCGTGATATTGAGGTGCCAGCC
Enoyl-hydratase isomerase family protein	ECH	CU989620	Fatty acid oxidation	GCAAATTTTACAGCAATGGCATAG	GCCTCCAGAACAACTCAACCA
Enolase	Enolase	CU986328	Energy metabolism (glycolysis)	CCAGCCCTTCAGTCAGATGTG	GCCCCATCTCCTCCTAACG
Adipolipin	Lipstor	CU996665	Fatty acid strorage	AGAAGACCAAGGAAAGCAACCA	TGATGCTGGATTCGGCTAGA
Phosphatidylcholine transferase	Pctrans	CU997534	Lipids class remodelling	CCTCACCACAGACGGCAAA	ACACCAGTCTTAGCACCCCAGTT
Phospholipase a2 receptor 1	PLA2	CU994900	Eicosanoides synthesis	CAATAACTTCAATTCTCCGACCAA	TTCGCAGTGTTTGATTTTCCATA
Phospholipase delta 1	PLD1	CU993057	Lipids class remodelling	GCCATCATCCGATTCGTTGT	CCCTCTTGGATTGAATGGAATG
Phosphatidylserine decarboxylase	Psdec	CU990331	Lipids class remodelling	GGACTCTACGTCTGGATGTTCGA	ATACCGTCTGTCACTTCACCAAAAT
ATP synthase f0 subunit 6	AS6	EE677700	Energy metabolism (electron chain transport)	ATGCCAAGCATGTTCTACAGAGT	GCAAAGGATCGCTCCTACCAAAGC
Cytochrome c oxidase subunit i	CCOi	FP001743	Energy metabolism (electron chain transport)	TTCCAGTCTCAACGGTCCTTTC	GCGTAAGCCAGGTTGGTTTCTAT
Citrate synthase	CS-G	CG1753	Energy metabolism (acid citric cycle)	CCGCGCCGGGACCTCCGTCGGTGTTGTAG	TTCGTCGGACACAGAGTCTCCCAATTCTC
Glutamine Synthetase	GS	CG1753	Amino acid synthesis	ACGGAGGTTGACGGGACTT	GCTGGCACCACGATTGG
Mitochondrial nadh:ubiquinone oxidoreductase	NADHox	CU999020	Energy metabolism (electron chain transport)	GATGGCAGAAAAGGATAGAATGGT	TCATCAGGTCCTCCTCCAACTC
Catalase	CAT-G	CU996492	Antioxidant defenses	GGAGGTGCCCCGAACTATTT	TCTTCATCCGCCGAGTTGTAG
Superoxide dismutase extracellular	ecSOD	CU999489	Antioxidant defenses	AGAGAATCCTGAGCTACAGC	TGAGCAAAACTCTCTACAAGC
Glutathione peroxidase 3	GPX3	CU994955	Antioxidant defenses	CCGTTGCTCCCTCGCTTT	AAGATGGCGGCTGATTGC
Glutathione peroxidase 5	GPX5	CU988021	Antioxidant defenses	CGTTCGGCCCGGTAGTG	GCCGTTGACATCGCCTATTC
Glutathione reductase	GR-G	FP000015	Antioxidant defenses	GCCACCAAGGCCCAGTT	AGATTTTCGGCGGAGTGTCA
Heat Shock protein 70	HSP70	CX069205	Cytoprotection (chaperon protein)	ATGAGTAAACACCAACAGGCCATCGG	AAGATAGTGTTCGTAGGGTTCATGGC
Metallothionein	MT	CU998632	Cytoprotection	GAGGAGAAACATCAAGACTAAGAAAACA	ACACGAATCAGAGCAGACACATG
Peroxiredoxin 4	PRDX4	FP005664	Antioxidant defenses	CAGGGCTGCTGATGATGACA	GGTGCAGGCTTGGAAATGA
Peroxiredoxin 5	PRDX5	CU986700	Antioxidant defenses	GCTGTGGATTTGTTTGAGAAGGAT	TGAGCAACCAGGAGTGAAAGC
Peroxiredoxin 6	PRDX6	CU984218	Antioxidant defenses	ACTCCCATACCCCATCATTTCC	GGATCAACCATGCCCAGTTT
Pernin	PRN	CD526735	Cytoprotection (metals chelator)	CTCCTGATCATGCTGAACCT	GCTGGCACCACGATTGG GATCATGTTTGTTCGGTCATC
Superoxide dismutase	SOD-G	FP005332	Antioxidant defenses	GAAGACGGTGTCGCCAAAA	GGCCGGCCAAGTCGAT

### Microbiological analyses

#### RT PCR-DGGE

The bacterial populations in seawater were analyzed after RNA extraction followed by reverse transcription (RT) RNA and then amplification of partial 16S rRNA gene by polymerase chain reaction (PCR). The different sequences were separated by denaturing gradient gel electrophoresis (DGGE) resulting in a fingerprint of active bacteria. Analyses targeting rRNA are usually expected to be more relevant than those targeting bacterial DNA because metabolically active cells contain a higher level of intracellular 16S rRNA than quiescent cells. The RNA from water, microalgal food, *V. coralliilyticus* and larvae was extracted then reverse transcribed according to protocols described above in the RNA extraction and real-time PCR section. PCR was performed on formed cDNA using 16S rDNA gene primer sets (341f-GC: 5′-GC-clamp-CCTTACGGGAGGCAGCA-3′ and 518r: 5′-ATTACCGCGGCTGCTGG-3′). PCR products were loaded onto 8% (w/v) polyacrylamide gel with a denaturing gradient ranging from 40 to 80% for eubacterial primers. DGGE was carried out with the D-code DGGE system (Bio-Rad Laboratories, France). After electrophoresis, gels were stained with SYBR-gold and revealed at 540 nm using a fluorimager (typhoon 9400, Amersham). The number of bands was determined visually for each sample. A similarity matrix using Jaccard's distance index (S_jaccard_) was used to compare the fingerprints. The Jaccard index was calculated as S_jaccard_ = N_AB_/(N_A_+N_B_ − N_AB_), where N_AB_ is the number of similar bands between samples A and B; N_A or B_ is the sum of all bands in sample A or B. Specific DGGE bands chosen by comparison with bacterial controls were excised from gels in order to detect *V. coralliilyticus* during the experiment. The excised bands were amplified with 341f without the GC-clamp and 518r. The amplified products were then cloned into PCR II plasmids using the TA cloning kit and transformed into *Escherichia coli* DH5α (Fisher Scientific SA, France) according to the manufacturer's instructions. The DNA sequencing was done using the SP6 promoter primer using capillary **ABI3130 XL** sequencer (Applied Biosystems, Foster City, CA, USA). Sequences were analyzed using the Bioedit software and were compared to the GenBank database using the online software and the Basic Local Alignment Search Tool algorithm (BLAST). Details of PCR-DGGE and sequencing methods were described in Azandegbe *et al.* [36].

#### Flow cytometry

To complement DGGE analyses, the total bacterial load was also measured by flow cytometry [37]. Before freezing, 1% glutaraldehyde was added on seawater samples. Frozen water samples were then quickly thawed in a 30°C water bath and were stained with SYBR Green I (Invitrogen). Bacteria were counted with an Epics Altra flow cytometer (Beckman Coulter) fitted with a 488 nm laser operated at 15 mW. The green fluorescence of nucleic-acid–bound SYBR Green I was measured at 525±5 nm. The analyzed sample volume was determined from the change in mass corrected for a dead volume (the water volume taken from the sample tube but not yet counted when data acquisition is stopped). The cytograms obtained were analyzed using Expo32 v1.2b software (Beckman Coulter).

### Statistical analyses

Analyses for growth and feeding activity were carried out using the SAS® Software system (8.2). The significance value for all analyses was set at p < 0.05. PROC GLM (two-way ANOVA) was used to estimate the effect of bacterial challenge, experiment time, and their interaction. Where differences were detected, LSMEANS (t-test) tests for multiple comparisons were used to determine which means were significantly different. Residuals were graphically assessed for normality using the PROC PLOT function coupled with the univariate procedure (PROC UNIVARIATE). Homogeneity was tested using the O'Brien test. Significant differences (p < 0.05) on survival rate between challenged and unchallenged ones at each time of the experiment were evaluated using Khi square tests (chisq.test script on R-language) performed on counts of dead and alive larvae.

A canonical redundancy analysis (RDA) was performed as a form of MANOVA, as proposed by Legendre and Anderson [38]. This method allows an estimation of the fraction of variation in 117 response variables (biochemical, physiological, and molecular parameters) attributable to the explanatory variables (time, bacterial challenge, and their interaction). This method, which is valid for small samples and non-normally distributed data, can be described as a serie of multiple regressions followed by a principal component analysis where each response variable, Y, is regressed on the matrix corresponding to the explanatory variables, X [39]. More precisely, matrices for challenge and time effect were coded using orthogonal dummy variables, and the interaction matrix was obtained by computing the product of the dummy variables representing the challenge and time effects. Each explanatory variable was individually tested with the others as covariables (matrix Y) to remove the variance explained by these other factors. An overall test of significance of the canonical relationship (999 permutations) and an ordination biplot (Z-plot type) were generated from the RDA results using plotRDA function [39]. The biplot displays both differences between batches of larvae and correlations between the response and explanatory variables. The main features of a correlation biplot are the following: (1) Projecting an object at right angle on a response (X) or explanatory (Y) variable approximates the value of the object along that variable. (2) The angles between variables (from sets X and Y) in the biplot reflect their correlations.

The first RDA was performed on the whole data set (i.e. 117 variables). A second set of RDA was also performed to estimate the challenge effect at each time and the time effect in challenged and unchallenged larvae. For this second set of RDA, four explanatory variables were built: “Chal24” and “Chal48” for the challenge effect at 24 h and 48 h, respectively, and “Time Chal” and “Time Unchal” for the time effect in challenged and unchallenged larvae. RDA results related to this second set were regrouped in two biplots, one illustrating the challenge effect and a second showing temporal change. Multiple comparisons (t-tests) were used to test the significance of differences observed in RDA. Due to the high number of variables, only those showing a significant difference for challenge and/or time effect in tests for multiple comparisons were included in the corresponding biplots. Concerning lipid profiles, although all fatty acids were analyzed, only essential fatty acid and sum of fatty acid classes were presented and discussed because of their importance in larval development and immunity (for overall fatty acid profiles see Tables S2.2 and S2.3 in File S2).

The R-language package rdaTest, available on the Web page http://www.bio.umontreal.ca/legendre/indexEn.html, was used to compute the canonical analyses (RDA) and produce biplot graphs. A posteriori comparison tests (LSMEANS, 8.2 SAS® software) were used to estimate the significance of differences observed in RDA. If normality precepts were not observed, data were LOG transformed.

## Results

### Larval performance

**Figure 1:**
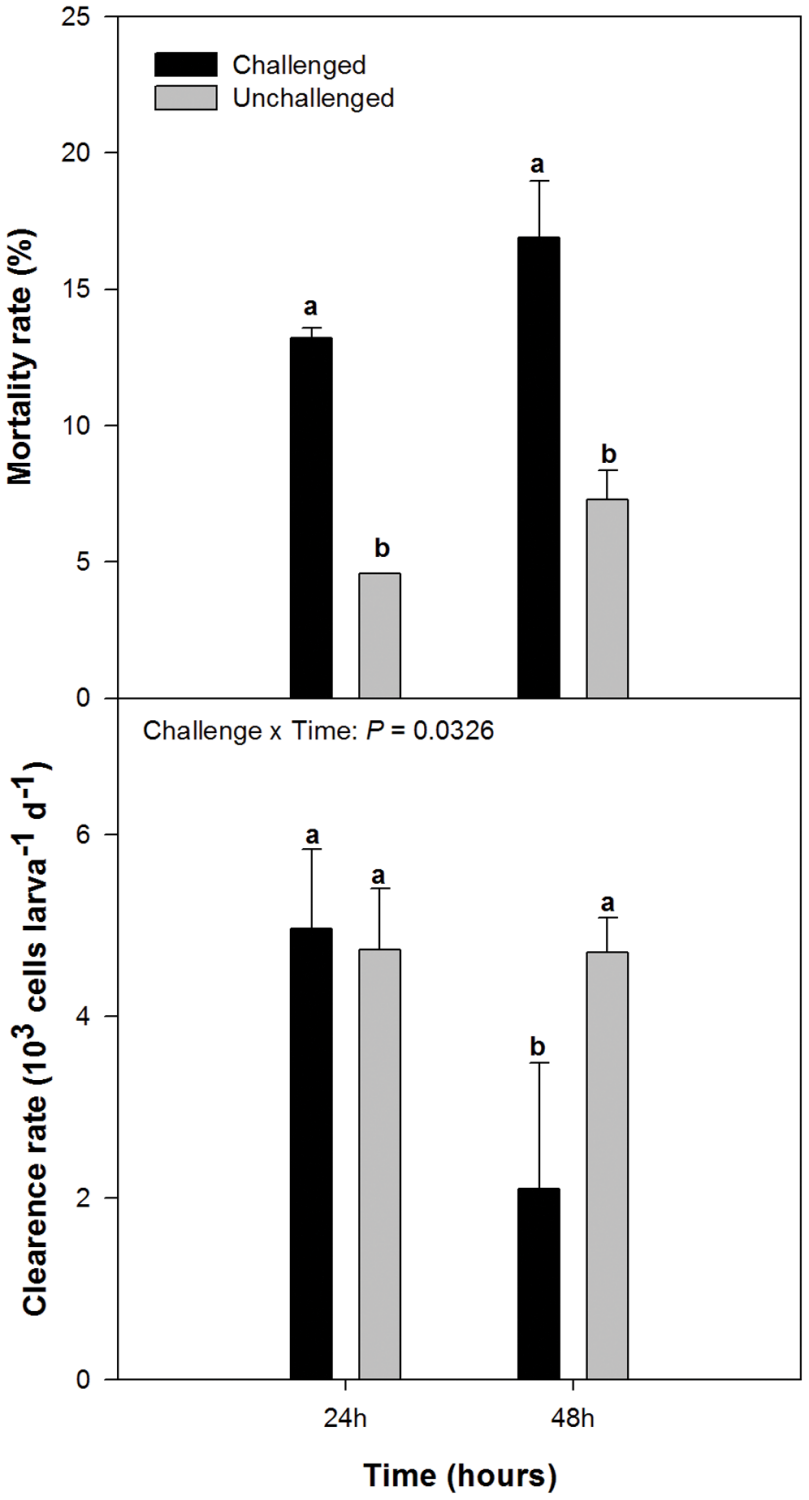
Mortality and clearance rate for Pacific oyster *Crassostrea gigas* larvae as a function of experiment duration and bacterial challenge. Larvae were challenged with *Vibrio coralliilyticus* for a period of 24 h or 48 h. Data from different treatments were pooled when this effect was not significant. Chal: challenged larvae; Unchal: unchallenged larvae. Data are means±SD of duplicate tanks. Different letters indicate significant differences.

Bacterial challenge affected directly larval survival with 13% and 17% of mortality rate in challenged larvae compared to 5% and 7% in control larvae after 24 h and 48 h, respectively (Figure 1). Significant interactions between time and bacterial challenge were observed for feeding activity. Feeding activity decreased significantly after 48 h of pathogen exposure, down to 67% less than in unchallenged larvae (Figure 1). Concerning growth, no significant effects of the challenge were noted in shell length after bacterial challenge (*p*-value: challenge = 0.407, time = 0.066, interaction = 0.731) with an average growth rate of 4.9±0.8 µm day^−1^ (over 48 h in comparison with the size of reference larvae) for both larvae batches.

### Bacterial community analyses

**Figure 2:**
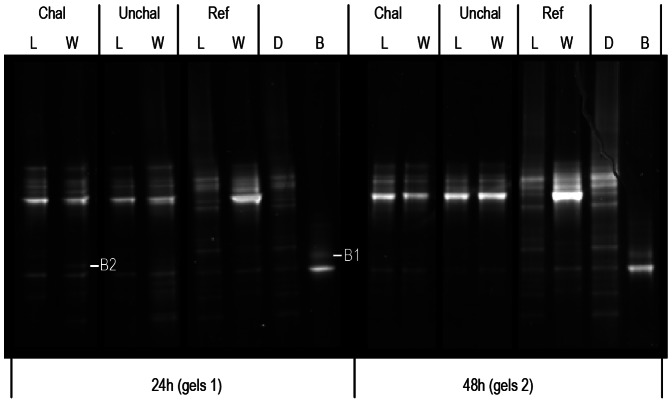
DGGE gels performed with 16 s cDNA extracted from larvae (L) and surrounding water (W). Profiles were compared with (Chal) or without (Unchal) bacterial challenge and after 24 h and 48 h of experiment. The cDNA from reference sample (Ref), diet (D) and bacterial control (B) was used as ladder. Due to their high similarity, cDNA from duplicate samples were pooled. B1 and 2 correspond to the retrieved bands used to *Vibrio coralliilyticus* detection by sequencing analysis.

Two bands were excised from DGGE gels used for *V. coralliilyticus* detection (Figure 2). After DNA sequencing, the BLAST treatment confirmed that the sequences obtained were highly similar to *V. coralliilyticus* 16S rRNA gene (see Tables S1.1 and S1.2 in File S1 for BLAST results and Figure S1.1 in File S1 for sequence alignment). In seawater, *V. coralliilyticus* was detected only 24 h after the beginning of the challenge. The Jaccard index matrix showed similarities between duplicates greater than 90%. Furthermore, in addition to the presence/absence of bands associated with *V. coralliilyticus*, high similarity (above 75%) was found between the DGGE profiles of challenged and unchallenged larvae at 24 h and 48 h. These results showed that pathogen inoculation induced limited changes in the active bacteria environment throughout the experiment. Flow cytometry analyses revealed a significant decline in the bacterial load (BL) after 48 h, independently of bacterial challenge (Time: *p-*value =  0.046; BL_24__h_: 1.3×10^5^±0.1 bacteria mL^−1^; BL_48__h_: 0.9×10^5^±0.1 bacteria mL^−1^).

### Redundancy analysis

The first set of RDA performed on the whole data set (i.e. 117 variables) shown that the overall variance was explained significantly by time, bacterial challenge and their interaction (challenge: *R^2^*: 0.26, *F*: 3.79, *p*: 0.03; time: *R^2^*: 0.26, *F*: 4.22, *p*: 0.026; interaction: *R^2^*: 0.19, *F*: 2.80, *p*: 0.031). The significant interaction effect led us to test the effect of each factor separately in each class of the other factor. For this, the second set of RDA analyses was performed to estimate the challenge effect at each time and the time effect in challenged and unchallenged larvae. Multiple comparisons used to test the significance of differences observed in these RDA showed that 52 parameters varied significantly with bacterial challenge and 57 with time. The mean, standard deviation, and variance explained by the RDA modelas well as results of multiple comparisons for each response variable are summarized in Tables S2.1 to S2.5 of File S2. Because we investigated changes associated to pathogen exposure, only results about challenge effect were presented in this section but the biplot illustrating time effect was included in Figure S3.1 of File S3.

**Figure 3:**
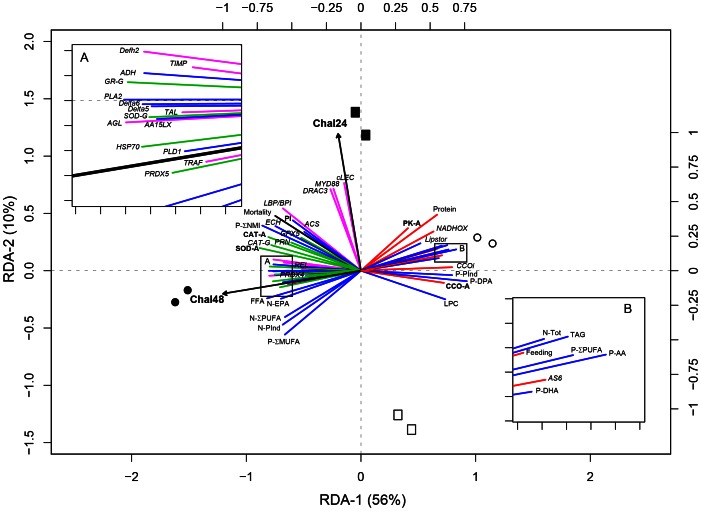
RDA ordination biplot representing the significant (p<0.05) challenge effect after 24 (Chal24) and 48 h (Chal48) as explanatory variables (arrows) on selected response variables (lines) related to energy metabolism (red), lipids (blue), cellular stress (green), and immunity (pink). Response variables related to larval performance and biochemical analysis were formatted in normal text, to enzymatic analysis in bold and to molecular analysis in italic. Black symbols: challenged larvae, white symbols: unchallenged larvae, squares: 24 h of exposure, circles: 48 h of exposure. Response variables abbreviations: -A, enzymatic activity; -G, gene expression; N-, neutral lipids; P-, polar lipids; Σ, sum of; cLEC, c-type lectin-1; AGL, a-agglutinin attachment subunit; LBP/BPI, LPS binding/bactericidal-permeability-increasing protein; MYD88, Myeloid differentiation primary response (88); TRAF, TNF receptor associated factor; REL, REL protein; DRAC3, drosophila rho GTPase 3; TAL, hematopoietic transcription factors; TIMP, tissue inhibitor metalloproteinase; Defh2, defensine 2; GR, glutathione reductase; SOD, superoxide dismutase; CAT, catalase; GPX5, glutathione peroxidase 5; PRDX4-5, peroxiredoxine 4 and 5; PRN, Pernin; HSP70, Heat shock protein 70; NADHOX, Mitochondrial nadh:ubiquinone oxidoreductase; CCOi, Cytochrome c oxidase subunit i; AS6, ATP synthase f0 subunit 6; PK, pyruvate kinase; CCO, cytochrome c oxidase; ADH, Acyl-CoA dehydrogenase; ECH, Enoyl-hydratase isomerase family protein; ACS, Acyl-CoA synthetase; Lipstor, Adipophilin; Delta5, Stearoyl-desaturase 5; Delta6, Fatty acid desaturase 2; PLA2, Phospholipase a2 receptor 1; PLD1, Phospholipase delta 1; AA15LX, Arachidonate 15-lipoxygenase; TOT, total lipids; PI, phosphatidylinositol; LPC, lysophosphatidylcholine; TAG, triacylglycerols; FFA, free fatty acids; AA, arachidonic acid; EPA, eicosapentaenoic acid; DPA, Docosapentaenoic acid; DHA, Docosahexaenoic acid; MUFA, monounsaturated fatty acids; PUFA, polyunsaturated fatty acids; NMI, non-methylene–interrupted fatty acids; Pind, peroxidation index; Protein, total proteins content; Feeding, feeding activity; Mortality, mortality rate.

RDA analyses performed on the 52 response variables showing a significant effect of challenge at 24 and/or 48 h (*R^2^ adj.*: 0.48, *F*: 4.22, *p*: 0.01) indicated that pathogen exposure explained 63% of the overall variance of these selected variables. The resulting biplot presented in figure 3 showed that physiological changes associated to pathogen exposure were principally observed after 48 h. Means, standard deviation and results of multiple comparisons for selected response variables associated to energetic metabolism, lipids, immunity and oxidative stress were regrouped in tables 2, 3 and 4 respectively. Response variables could be classified into groups of highly correlated variables in function of their response to pathogen exposure. In regard of the functional group studied, we found that the impact of *V. coralliilyticus* exposure on energy metabolism in larvae could be seen after 48 h by the lower relative expression of three genes related to energy production (CCOi, AS6, NADHOX) and by the lower activities of cytochrome c oxidase (CCO-A, 0.47±0.03 vs 0.62±0.02 pmol larva^−1^ min^−1^) and pyruvate kinase (PK-A, 6.05±0.41 vs 8.69±0.32 pmol larva^−1^ min^−1^) comparatively to uninfected animals.

**Table 2:** Summary of means (± STD) and statistics for parameters related to energetic metabolism.

			Mean±STD				Multiple comparisons			
Analyses	RV	Unity	24 h Chal	24 h Unchal	48 h Chal	48 h Unchal	24 h Chal	24 h Unchal	48 h Chal	48 h Unchal
Gene expression	AS6	Relative expression	0.86±0.09	0.67±0.32	1.26±0.73	9.60±2.92	b	b	b	a
	CCOi	Relative expression	0.83±0.1	0.61±0.12	0.76±0.21	2.72±0.09	b	b	b	a
	NADHox	Relative expression	1.10±0.19	0.63±0.3	0.89±0.03	2.27±0.67	b	b	b	a
Enzymatic activity	CCO-A	pmol larve^−^ min^−1^	0.45±0.05	0.49±0.04	0.47±0.03	0.62±0.02	b	b	b	a
	PK-A	pmol larve^−1^ min^−1^	5.13±0.62	3.22±0.06	6.05±0.41	8.69±0.32	b	c	b	a
Biochemical content	Protein	ng protein larva^−1^	40.9±1.57	37.86±0.33	38.01±0.21	43.43±2.49	ab	b	b	a

Multiple comparisons show the significant differences (letters) between each combination of treatments. Abbreviations: RV, response variable name in RDA biplot, 24 and 48 h chal, larvae challenged during 24 or 48 h; 24 and 48 h unchal, unchallenged larvae after 24 and 48 h. Response variables abbreviations: NADHOX, Mitochondrial nadh:ubiquinone oxidoreductase; CCOi, Cytochrome c oxidase subunit i; AS6, ATP synthase f0 subunit 6; PK-A, pyruvate kinase activity; CCO-A, cytochrome c oxidase activity; Protein, total proteins content.

**Table 3:** Summary of means (± STD) and statistics for parameters related to lipids.

			Mean±STD				Multiple comparisons			
Analyses	RV	Unity	24 h Chal	24 h Unchal	48 h Chal	48 h Unchal	24 h Chal	24 h Unchal	48 h Chal	48 h Unchal
Biochemical content	N-Tot	ng lipids larva^−1^	4.71±1.1	4.35±0.89	2.09±0.13	4.70±0.23	a	a	b	a
	FFA	mass %	2.53±0.02	3.07±0.15	6.51±0.08	2.16±0.03	c	b	a	d
	TAG	mass %	79.59±1.69	78.47±2.49	71.28±0.94	80.39±3.12	a	a	b	a
	N-EPA	mass %	4.72±0.31	4.90±0.01	5.89±0.05	4.83±0.25	b	b	a	b
	N-ΣPUFA	mass %	49.77±1.12	50.72±0.39	50.50±0.39	48.13±0.17	ab	a	a	b
	N-Pind	-	219±6	225±3	230±4	217±2	ab	a	a	b
	LPC	mass %	4.04±0.98	6.7±0.23	5.31±1.44	10.26±1.12	b	b	b	a
	PI	mass %	18.14±0.84	15.06±2.02	17.03±0.87	13.84±1.98	a	ab	a	b
	P-AA	mass %	2.43±0.09	2.33±0.01	2.12±0.09	3.86±0.12	b	b	b	a
	P-DPA	mass %	2.54±0.17	2.86±0.06	2.18±0.07	3.53±0.05	c	b	d	a
	P-DHA	mass %	19.64±0.69	19.65±0.01	19.95±0.01	22.97±1.15	b	b	b	a
	P-ΣNMI	mass %	10.91±0.09	8.14±0.51	11.62±0.22	7.53±0.25	a	b	a	b
	P-ΣMUFA	mass %	21.75±0.30	24.41±1.03	24.28±0.24	19.79±1.53	b	a	ab	b
	P-ΣPUFA	mass %	58.81±0.67	58.40±0.84	58.22±0.07	63.84±1.45	b	b	b	a
	P-Pind	-	292±7	301±6	293±1	333±12	b	b	b	a
Gene expression	AA15LX	Relative expression	2.19±0.57	1.37±0.35	8.76±3.08	1.69±0.47	b	b	a	b
	ADH	Relative expression	2.94±0.20	1.99±0.97	5.90±1.93	1.96±0.28	b	b	a	b
	Delta5	Relative expression	0.58±0.14	0.40±0.36	1.64±0.2	0.47±0.19	b	b	a	b
	Delta6	Relative expression	0.54±0.01	0.15±0.06	2.67±0.85	0.27±0.01	b	b	a	b
	ECH	Relative expression	2.89±1.38	0.55±0.22	4.09±0.38	0.67±0.39	a	b	a	b
	Lipstor	Relative expression	0.47±0.43	0.23±0.12	1.58±0.10	1.43±0.06	b	b	a	a
	PLA2	Relative expression	0.51±0.46	0.16±0.09	2.17±0.59	0.09±0.04	b	b	a	b
	PLD1	Relative expression	1.56±0.92	1.5±0.24	5.4±0.5	1.96±0.67	b	b	a	b

Multiple comparisons show the significant differences (letters) between each combination of treatments. Abbreviations: RV, response variable coding in RDA biplot, 24 and 48 h chal, larvae challenged during 24 or 48 h; 24 and 48 h unchal, unchallenged larvae after 24 and 48 h. Response variables abbreviations: N-, neutral lipids; P-, polar lipids; Σ, sum of; TOT, total lipids; PI, phosphatidylinositol; LPC, lysophosphatidylcholine; TAG, triacylglycerols; FFA, free fatty acids; AA, arachidonic acid; EPA, eicosapentaenoic acid; DPA, Docosapentaenoic acid; DHA, Docosahexaenoic acid; MUFA, monounsaturated fatty acids; PUFA, polyunsaturated fatty acids; NMI, non-methylene–interrupted fatty acids; Pind, peroxidation index; ADH, Acyl-CoA dehydrogenase; ECH, Enoyl-hydratase isomerase family protein; ACS, Acyl-CoA synthetase; Lipstor, Adipophilin; Delta5, Stearoyl-desaturase 5; Delta6, Fatty acid desaturase 2; PLA2, Phospholipase a2 receptor 1; PLD1, Phospholipase delta 1; AA15LX, Arachidonate 15-lipoxygenase.

**Table 4:** Summary of means (± STD) and statistics for parameters related to immunity and oxidative stress.

			Mean±STD				Multiple comparisons			
Analyses	RV	Unity	24 h Chal	24 h Unchal	48 h Chal	48 h Unchal	24 h Chal	24 h Unchal	48 h Chal	48 h Unchal
Gene expression	AGL	Relative expression	0.77±0.19	0.33±0.35	4.25±0.69	0.29±0.27	b	b	a	b
	CAT-G	Relative expression	3.04±0.40	0.65±0.07	7.45±0.70	1.72±1.06	b	b	a	b
	cLEC	Relative expression	3.44±0.52	0,01±0,01	0.09±0.03	0.01±0,01	a	b	b	b
	Defh2	Relative expression	0.60±0.26	0.14±0.04	1.81±0.49	0.30±0.03	b	b	a	b
	DRAC3	Relative expression	1.17±0.42	2.73±0.47	1.10±0.03	0.59±0.2	b	a	b	b
	GPX5	Relative expression	1.59±0.08	0.83±0.53	2.7±0.21	1.74±0.07	b	b	a	b
	GR-G	Relative expression	0.57±0.24	0.13±0.02	2.19±0.66	0.15±0.17	b	b	a	b
	HSP70	Relative expression	1.24±0.95	1.02±0.46	5.79±1.50	0.83±0.06	b	b	a	b
	LBP/BPI	Relative expression	8.28±2.34	1.50±0.34	8.86±4.31	2.14±0.9	a	b	a	ab
	MYD88	Relative expression	11.33±1.09	1.58±1.39	2.01±0.17	0.46±0.39	a	b	b	b
	PRDX4	Relative expression	0.45±0.3	0.36±0.26	2.79±0.14	1.59±0.25	c	c	a	b
	PRDX5	Relative expression	0.79±0.06	0.86±0.89	2.61±0.19	0.82±0.10	b	b	a	b
	PRN	Relative expression	0.65±0.1	0.09±0.04	1.64±0.30	0.67±0.21	b	c	a	b
	REL	Relative expression	0.96±0.59	0.63±0.21	3.61±0.61	1.81±0.31	b	b	a	b
	SOD-G	Relative expression	0.64±0.52	0.19±0.13	4.18±0.86	0.47±0.38	b	b	a	b
	TAL	Relative expression	0.37±0.28	0.18±0.05	1.81±0.73	0.37±0.06	b	b	a	b
	TIMP	Relative expression	1.55±0.39	0.35±0.11	6.03±1.58	1.68±0.71	b	b	a	b
	TRAF	Relative expression	0.45±0.05	0.52±0.09	3.52±0.59	0.95±0.85	b	b	a	b
Enzymatic activity	CAT-A	pmol larve^−1^ min^−1^	102.51±5.19	86.86±5.89	123.28±0.55	93.72±3.15	b	b	a	b
	SOD-A	pU larve^−1^ min^−1^	1947±328	1549±21	2212±133	1146±378	a	ab	a	b

Multiple comparisons show the significant differences (letters) between each combination of treatments. Abbreviations: RV, response variable coding in RDA biplot, 24 and 48 h chal, larvae challenged during 24 or 48 h; 24 and 48 h unchal, unchallenged larvae after 24 and 48 h. Response variables abbreviations: -A, enzymatic activity; -G, gene expression cLEC, c-type lectin-1; AGL, a-agglutinin attachment subunit; LBP/BPI, LPS binding/bactericidal-permeability-increasing protein; MYD88, Myeloid differentiation primary response (88); TRAF, TNF receptor associated factor; REL, REL protein; DRAC3, drosophila rho GTPase 3; TAL, hematopoietic transcription factors; TIMP, tissue inhibitor metalloproteinase; Defh2, defensine 2; GR, glutathione reductase; SOD, superoxide dismutase; CAT, catalase; GPX5, glutathione peroxidase 5; PRDX4-5, peroxiredoxine 4 and 5; PRN, Pernin; HSP70, Heat shock protein 70.

Apart from effects related to energy metabolism, bacterial challenge induced changes in lipid composition and protein content. After 48 h, challenged larvae had lower protein (38.01±0.21 vs 43.43±2.49 ng larva^−1^) and neutral lipid contents (2.1±0.1 vs 4.7±0.2 ng larva^−1^), and exhibited higher free fatty acids (FFA, 6.5±0.1 vs 2.2±0.1% of neutral lipids), lower proportions of triglycerides (TAG, 71±1 vs 80 ±3% of neutral lipids) and lysophosphatidylecoline (LPC, 5.3±1.4 vs 10.3±1.1% of polar lipids); phosphatidylinositol (PI) was the only lipid class that was always higher in challenged larvae (17.6±0.8 vs 14.5±1.9% of polar lipids). Interestingly, the lower neutral lipid content in infected larvae coincided with the down-expression of Lipstor, a gene involved in lipid storage. Neutral lipids from challenged larvae after 48 h were characterized by a higher peroxidation index (N-PInd, 230±4 vs 217±2) and higher proportions of polyunsaturated fatty acids (N-ΣPUFA, 50.5±0.4 vs 48.1±0.2% of neutral lipids), free fatty acids (FFA, 6.5±0.1 vs 2.2±0.1% of neutral lipids), and 20∶5(n-3) (N-EPA, 5.9±0.1 vs 4.8±0.3% of neutral lipids) compared to unexposed oysters. After 48 h of pathogen challenge, the polar fraction showed a lower peroxidation index (P-Pind, 293±1 vs 333±12); lower proportions of polyunsaturated fatty acids (P-ΣPUFA, 58.2±0.1 vs 63.8±1.5% of polar lipids), 20∶4(n-6) (P-AA, 2.1±0.1 vs 3.9±0.1% of polar lipids), 22∶6(n-3) (P-DHA, 20.0±0.1 vs 23.0±1.2% of polar lipids), and 22∶5(n-6) (P-DPA, 2.2±0.1 vs 3.5±0.1% of polar lipids); and higher amounts of monounsaturated fatty acids (P-ΣMUFA, 24.3±0.2 vs 19.8±1.5% of polar lipids). Challenged larvae also showed a higher proportion of non-methylene–interrupted fatty acids in the polar lipids (P-ΣNMI, 11.6±0.2 vs 7.5±0.3% of polar lipids) than unchallenged oysters. These changes in fatty acid composition were reflected at the molecular level by the over-expression of genes associated with lipid metabolism in challenged larvae after 24 h for ACS and ECH, and after 48 h for PLA2, PLD1, AA15LX, ACDH, Delta5, and Delta6.

Pathogen challenge induced the activation of antioxidant defenses, as revealed by the higher transcript abundance of antioxidant enzymes measured after 24 h of exposure for GPX5 and after 48 h for GR-G, SOD-G, CAT-G, PRDX4, and PRDX5. This over-expression of catalase (CAT-G) and superoxide dismutase (SOD-G) in challenged larvae was reflected at the physiological level by the higher activities of the corresponding enzymes (CAT-A, 123±1 vs 94±3 pmol larva^−1^ min^−1^;SOD-A, 2212±133 vs 1146±378 pU larva^−1^ min^−1^). We recorded higher gene expression of proteins associated with cellular stress (HSP70, PRN) simultaneous to the activation of antioxidant defenses. Finally, immune response activation was observed 24 h after bacterial stimulation by the over-expression of four genes related to the immune reaction (MYD88, cLEC, DRAC3, LBP/BPI) followed at 48 h by the activation of six others (Defh2, TIMP, TAL, TRAF, REL, AGL).

## Discussion

### Relevance of our bacterial challenge

To study the host response to a pathogenic bacterial challenge, a good balance must be reached between the absence of significant effects or, alternatively, massive mortality leading to rapid degradation of the studied host and bacterial proliferation on dead tissues. The timing of sampling and inoculation dose are key parameters allowing to document such responses. In our study, we recorded a limited but significant increase of mortality in larvae exposed to *V. coralliilyticus.* It indicated that the inoculation with 10^4^ bacteria per mL induced a significant impact on *C. gigas* larvae after 24 to 48 h, but did not lead to a septicaemia response or a massive mortality. The sequencing of RT PCR-DGGE bands revealed the presence of 16S cDNA from *V. coralliilyticus* in the larval culture water after 24 h of challenge, confirming that *V. coralliilyticus* was metabolically active at this time. After 48 h of challenge, its disappearance could be due to the detection limit of the PCR-DGGE technique. Flow cytometry analyses showed that the bacterial load slightly declined during the experiment, suggesting that no bacterial proliferation occurred after 48 h, and therefore that the concentration of active *V. coralliilyticus* dropped below the detection limit after 48 h. The reason for this declined bacterial load could be explained by an increase of bacterial mortality or a decrease of bacterial activity for *V. Coralliilyticus,* which could be associated with the agglutinating and clearance activity of larvae against pathogen as suggested by the activation of immune parameters described below. Finally, our analysis of the similarity index based on DGGE profiles demonstrated that regardless of the presence or absence of *V. coralliilyticus*, the composition of the remaining active bacteria surrounding the larvae and those associated with them was similar independently of pathogen stimulation.

### Physiological response to bacterial infection

#### Immune response

Bivalve larvae lack specific immune responses and immunological memory. Therefore they rely totally on their innate immune system to overcome diseases [40]. The innate immune system of bivalves employs cellular and humoral components that operate in a coordinated way to provide protection from pathogens [1], [2]. The first action of the oyster's immune system when challenged by micro-organisms is the recognition of these foreign organisms. This is achieved by oyster immune receptors interacting with a highly conserved set of molecular structural motifs present on the surface of micro-organisms that are absent from host cells, allowing the discrimination between self and non-self. In our study, four genes were selected in relation with non-self-recognition (GAL8, cLEC, AGL, LBP/BPI). While GAL8 remained unchanged during our bacterial challenge, cLEC and LBP/BPI were activated after 24 h and AGL after 48 h. The increased cLEC and AGL expressions observed in this study are related to genes coding for a c-type lectin 1 and for an a-agglutinin attachment subunit respectively. Agglutinins and lectins are glycoproteins specialized in the recognition of specific pathogen-associated carbohydrate structures and cause the agglutination of cells, opsonisation, the promotion of cellular adhesion, and the mediation of the innate immune response [41], [42]. Lectin expression in adult oyster has been shown to increase after bacterial exposure [5], [43]. Interestingly, Tirape *et al.* [7] observed that the expression of galectin 8 (GAL8), another lectin family, was not activated after bacterial challenge in juvenile *C. gigas*, in accordance with our results. Firstly indentified as a LPS binding proteins (LBP) coding gene [44], LPB/BPI has recently been associated to a bactericidal-permeability-increasing protein (BPI) [45]. BPI is specifically active against gram-negative bacteria (such as *V. coralliilyticus*); it increases the permeability of the bacterial membranes allowing the opsonisation of bacteria [45]. The activation of LBP/BPI by bacterial challenge at larval stage resembles what was already observed in adults [45], [46]. Thus this protein may participate to the first line of defense early during the development as suggested by Tirape *et al.* [7].

Upon successful recognition of pathogenic micro-organisms, signaling cascades are triggered for the initiation of immune responses and for the transcription of inducible immune-related proteins. Over the past decade, the Rel and nuclear factor-κB (Rel/NF-κB) signal transduction pathway appeared to be a key component of the bivalve immune response inducing the transcription of numerous genes involved in immune function and inflammation in adult [3], [5], [47]. In our study, we observed a higher expression of three genes related to this signalling pathway (REL, MYD88 TRAF) in challenged larvae, reinforcing the hypothesis of the occurrence of this immune component at larval stage in *C. gigas* as suggested by Tirape *et al.* [7]. The over-expression of TIMP (Tissue Inhibitors of Metalloproteinase) in challenged larvae after 48 h could be related to activation of the Rel/NF-κB pathway. Indeed, as described in Montagnani *et al.* [48], the presence of three κB-motifs in the TIMP promoter suggests that TIMP gene expression might be controlled by the oyster's Rel/NF-κB pathway. Its function in the immune response might be linked to its ability to inhibit protease and metalloproteinase, two key components of bacterial infectivity [49]. It has been demonstrated that TIMP mRNA accumulation in haemocytes was induced following the injection of proteases produced by pathogenic Gram-negative bacteria [48] or after *Vibrio splendidus* infection [5]. These observations were in accordance with our results, where the stimulation by the Gram-negative *V. coralliilyticus* induced the over-expression of TIMP after 48 h of bacterial challenge. Although it has not yet been demonstrated in bivalves, the Rel/NF-κB pathway is also known to activate antimicrobial peptides synthesis in invertebrates, especially insects [50], [51]. Antimicrobial peptides are amongst the most important effectors of innate immunity and display a variety of action mechanisms, killing microbes by membrane disruption or by altering metabolic processes related to the synthesis of cell walls [52]. In our study, after 48 h of pathogen challenge, we observed an increase of Defh2 transcript level, a gene coding for an antimicrobial peptide family, the defensins. Defensins have been widely studied in bivalves, including adult Pacific oysters [53], [54], [55] and their activation after bacterial challenge has been well demonstrated [46], [55]. The activation of defensin synthesis after a bacterial challenge in veliger larvae suggests that antimicrobial peptide defences prevail earlier in oyster development.

Accurate immune response involves haemocytes differentiation (hematopoiesis) allowing haemocytes proliferation around the infection site. In this study, the activation of haemocytes differentiation was revealed by increases in TAL and DRAC3 transcript levels after bacterial challenge. Tirape *et al.* [7] used these two genes to demonstrate that hematopoiesis is present earlier during larval development in *C. gigas*. In the same study, an increase of Cg-tal and DRAC3 after bacterial challenge was already observed corroborating our results.

#### Oxidative stress

The final elimination of infecting bacteria is presumed to be carried out in phagolysosome involving various cytotoxic reactions, such as the release of lysosomal enzymes and the production of reactive oxygen species (ROS). The generation of these various free radicals during intracellular destruction of phagocytized bacterial material induces the activation of antioxidant defenses to avoid the peroxidation of host tissues. In our results, challenged larvae showed higher activities of catalase (CAT-A) and superoxide dismutase (SOD-A), two key enzymes implicated in antioxidant defenses, indicating their activation after pathogen stimulation. This activation was also reflected at the molecular level by the over-expression in infected larvae of the antioxidant related genes GPX5, GR-G, CAT-G, SOD-G, PRDX-4, and PRDX-5 related to glutathione peroxidase (family 5), glutathione reductase, catalase, superoxide dismutase, and peroxiredoxin (family 4 and 5), respectively. These results coincided with previous work in American oyster larvae [11] and adult pacific oysters [56] in which higher expression levels of antioxidant enzymes were observed during pathogenic conditions. Interestingly, in our study, no increase in lipid peroxidation was observed in infected larvae suggesting that they developed accurate antioxidant defences activation to avoid tissue peroxidation.

Besides antioxidant defences, larvae possess various cytoprotective processes to avoid cell degradation by ROS. The expression of two genes related to these processes (HSP70 and PRN) were up-regulated after 48 h of bacterial challenge. Heat shock proteins (HSP) are stress-response proteins implicated in cytoprotection that act as molecular chaperones, binding to damaged or misfolded polypeptides, either facilitating their repair or targeting irreparably damaged proteins for degradation [57]. Increases of HSP expression have been observed after *vibrio* infection in adult pacific oysters [3], [5]. In *C. virginica*, the expression of HSP70 has been shown to increase in haemocytes with increasing intensities of *Perkinsu*s infection [58] and after massive mortality events of larval stages [11]. Pernin (PRN) also called cavortin, is a glycosylated protein known to participate in the binding of divalent metal cations, suggesting that PRN functions as a metal chelator or a chaperone. In addition, PRN acts as a serine protease inhibitor and has a sequence clearly homologous to the active-site domain of Cu–Zn SODs (superoxide dismutases) [59]. Tanguy *et al*. [60] identified a gene coding for a PRN among *C. virginica* haemocytes and observed an increase of mRNA expression after 45 days of *P. marinus* exposure. Moreover, Huvet *et al*.[61] suggested that PRN protects cell hosts against the reactive oxygen intermediate (ROI) in *C. gigas* inoculated with *Vibrio splendidus*. The over-expression of HSP70 and PRN in challenged larvae indicates that these proteins could play similar functions in cytoprotection and immunity, as it has been suggested in *C. virginica* larvae [11].

#### Energetic metabolism

To sustain immune, antioxidant and cytoprotection processes, larvae needed to allocate a part of their energy retrieved from feeding and/or energetic reserves. That is why we investigated the impact of bacterial infection on the components of energetic metabolism. The first evidence of this impact is the decline of feeding activity in challenged larvae after 48 h of exposure, as already observed during disease events in American oyster larvae [15]. This decline in food intake seemed to affect the ATP production, as illustrated by the lower activity of pyruvate kinase (PK-A) and cytochrome c oxidase (CCO-A), two enzymes involved in glycolysis and the respiratory chain complex respectively. In addition to these enzymes, this metabolic depression was also reflected at the molecular level as demonstrated by the lower gene expressions of cytochrome c subunit i (CCOi, respiratory chain complex IV), NADH ubiquinone:oxidoreductase (NADHox, respiratory chain complex I), and ATP synthase subunit 6 (AS6, respiratory chain complex V) in infected larvae. The metabolic depression associated to feeding declines was demonstrated in Manila clams juveniles (*Ruditapes philippinarum*) infected by brown ring disease [62] and in *C. virginica* larvae submitted to a massive mortality event [15]. The decline of food intake coincided with lower triglyceride (TAG) and protein content, the two main energetic reserves in bivalve larvae. The higher concentrations of free fatty acids (FFA) in challenged larvae after 48 h may be related to TAG catabolism. Indeed, FFA usually results from TAG degradation by lipase [63], [64].*_ENREF_81* TAG catabolism in challenged larvae could also be induced by the over-expression of acyl-CoA synthetase (ACS), acyl-CoA dehydrogenase (ACDH), and enoyl-CoA hydratase (ECH), three key enzymes of β-oxidation. The fatty acids stored in TAG are broken down sequentially through β-oxidation to yield acetyl-CoA, which can then be introduced into the Krebs cycle to produce energy [65], [66]. The β-oxidation has been little studied in marine invertebrates [67] and to our knowledge our study is the first to hypothesize the activation of this metabolic pathway in challenged bivalve larvae. Beside the β-oxidation activation, the lower ability for challenged larvae to accumulate lipids was observed with the down-expression of adipophilin (Lipstor), a protein involved in lipid droplet formation and triglyceride accumulation [68]. The decline of food intake and the utilization of energetic substrates could provoke a weakening of larvae if the infection last longer. It could in turn limit the energy availability for larval development and/or the activation of accurate immune, antioxidant and cytoprotection processes as suggested in American oyster larvae incurring massive mortality [11], [15]. In this context, converting energetic substrates after 48 h of experiment to energy equivalents with values of 24.0, 39.5 and 17.5 kJ g^−1^ for protein, lipid and carbohydrate respectively [69] yields a total energy content of 1442±14 µJ larva^−1^ for challenged larvae and 1679±88 µJ larva^−1^ for unchallenged ones. These results confirm that energy availability for metabolic requirements and development was lower in challenged larvae.

#### Structural lipids remodeling

The bacterial infection seemed to affect also structural lipid composition that contained higher PI and lower LPC in challenged larvae than control larvae. These changes could be linked to the utilization of derivates in immune response signalling. Indeed, it has been shown that phosphatidylinositol 3,4,5-triphosphate (PI3) generated by phosphatidylinositol-3 kinase (PI3K) is a crucial signal transducing element that regulates communication across the plasma membrane, especially during the immune response [70], [71]. Although this pathway was suggested to hold an important role in the immune response of molluscs [72], the effect on PI content has never been demonstrated and would necessitate further investigation.

Many studies in vertebrates have revealed that LPC could play an important part in immune response transduction. Indeed, LPC activates several second messengers in vertebrates, including extracellular signal-regulated kinases, mitogen-activated protein kinase (MAPK), phosphoinositide-3-kinase, Ca^2+^, adenylate cyclase (AC), Rho protein, and platelet-activating factor (PAF), all of which are known to regulate the inflammatory response [73], [74], [75]. However, the occurrence of these immune signalling processes remains largely unknown in marine invertebrates and needs further investigation.

Concerning fatty acids, changes in polar lipid composition were observed in challenged larvae. Unlike unchallenged larvae, challenged larvae did not accumulate major C_20_ PUFA (DHA, DPA, AA) after 48 h, as it is usually observed during development of bivalve larvae [23], [76]. Moreover, the level of the naturally biosynthesized NMI fatty acid (essentially 22∶2 NMI) was always higher in challenged larvae. All of these elements seem to indicate that challenged larvae, probably because their lower feeding activity, were unable to extract sufficient essential fatty acids from their diet and compensated for this lack in major PUFA by accumulating NMI fatty acids. Indeed, an increase in 22∶2 NMI may compensate for the decrease in other long-chain PUFA such as DHA [77]. Moreover, a higher NMI content in cell membranes could balance the decrease in the peroxidation index attributed to oxidative stress that may occur during infection as suggested under pathological condition in adult *C. gigas* by Pernet *et al.* [29]. This hypothesis is supported by the fact that NMI fatty acids are more resistant to oxidation than other PUFA [78]. It was also suggested that since NMI fatty acids are usually present in the outer membrane of mollusks, they may increase resistance to attack by microbial lipases [79].

Fatty acid remodeling after pathogen stimulation was also suggested by the activation of two genes coding for fatty acid 5 and 6 desaturases. The physiological role of delta 5 desaturase in marine invertebrates has not been extensively studied so far. Nevertheless, previous studies demonstrated that delta 5 desaturase plays an important role in the biosynthesis of NMI fatty acids [77], [80]. Moreover, the delta 5 and delta 6 desaturases govern the rate-limiting steps in the biosynthesis of long-chain PUFAs. Although it is well demonstrated in fish species [81], [82], the ability to synthesize long chain PUFAs *de novo* has not been clearly demonstrated in bivalves [77] and needs further investigation.

The activation of desaturases coincided with the over-expression of three other genes involved in lipid metabolism, AA15LX, PLA2, and PLD1. Phospholipase A2 (PLA2) is implicated in diverse cellular processes, but perhaps the most notable function of PLA2 is its ability to initiate the inflammatory response through eicosanoids synthesis. Following its production by PLA2, AA (or EPA) is metabolized through the cyclooxygenase (COX) and lipoxygenase (LX) pathways to yield prostaglandins and leukotrienes respectively [83]. AA15 lypoxygenase (AA15LX) was directly involved in the leukotrien transformation of AA to 15(S)-hydroxyeicosatetraenoic acid (15-HETE) [84], [85]. Interestingly, the activation of PLA2 and AA15LX could explain the AA decline in polar lipids of infected larvae after 48 h. The activation of the eicosanoids pathway through phospholipase and lipoxigenase activation during immune response was well demonstrated in insects [22], [86], and few references concerning eicosanoids production in *C. gigas* exist [87], [88]. Although it has not been formally demonstrated in bivalves, the Rel/NF-κB pathway is known to activate phospholipase A2 in invertebrates, especially insects [89], [90] as well as the AA15 lipoxigenase in vertebrate [91]. Our results for genes associated with PLA2, AA15LX, and Rel/NF-κB suggest that this situation could prevail in *C. gigas* larvae after pathogen exposure. This hypothesis, however, needs further support. Phospholipase D1 (PLD1) is implicated in several important cellular functions and is believed to have a signalling role in lysophosphatidic acid (LPA) immune pathway [92], [93]. Indeed, LPA may be synthesized from LPC produced by the PLA2 transformation of phosphatidylcholine (PC), or by the PLA2-catalyzed deacylation of phosphatidic acid (PA) generated by PLD1 [75]. Considering the lower LPC content and the activation of PLA2 and PLD1 in challenged larvae, we can hypothesize an activation of LPA pathway components after pathogen stimulation but here again the hypothesis requires additional experiments for validation.

#### Time-response of oyster larvae to bacterial infection

Finally, one of the most interesting aspects of this study is the possibility to compare physiological response at two times on a short term period (24 and 48 h). Although six genes related to immunity (LPB/BPI, DRAC3, MYD88 and cLEC), antioxidant defense (GPX5) and cytoprotection (PRN) were up-regulated after 24 h, a large part of the significant differences related to these processes was measured after 48 h of challenge. These results contrast with those found in adult oysters by De Decker and Saulnier [94] i.e. an up-expression of immune genes 2 h after *vibrio* injection. We think that this difference in time-response could be due to the bacterial challenge protocol (balneation vs intramuscular injection) and the pathogen concentration (10^4^ vs 10^8^ cells mL^−1^). However, the lack of sampling before 24 h of challenge implies that we didn't exclude the activation of immune parameters early during infection.

The activation of β-oxidation preceded the feeding decline, the consumption of energetic substrates and the metabolic depression It suggests that there is an increase of energy demand before 48 h probably to sustain the immune response initiation. However, no delay was observable between changes in metabolic enzymes activities and the regulation of related genes. Similar observations were made between the activity of antioxidant enzymes and the expression of associated genes. Concerning structural lipids, the activation of desaturases, phospholipases and lipoxigenase coincided with fatty acid remodelling in polar lipids. These elements illustrated that larvae responded quickly by deep changes at biochemical and physiological levels.

## Conclusion

**Figure 4:**
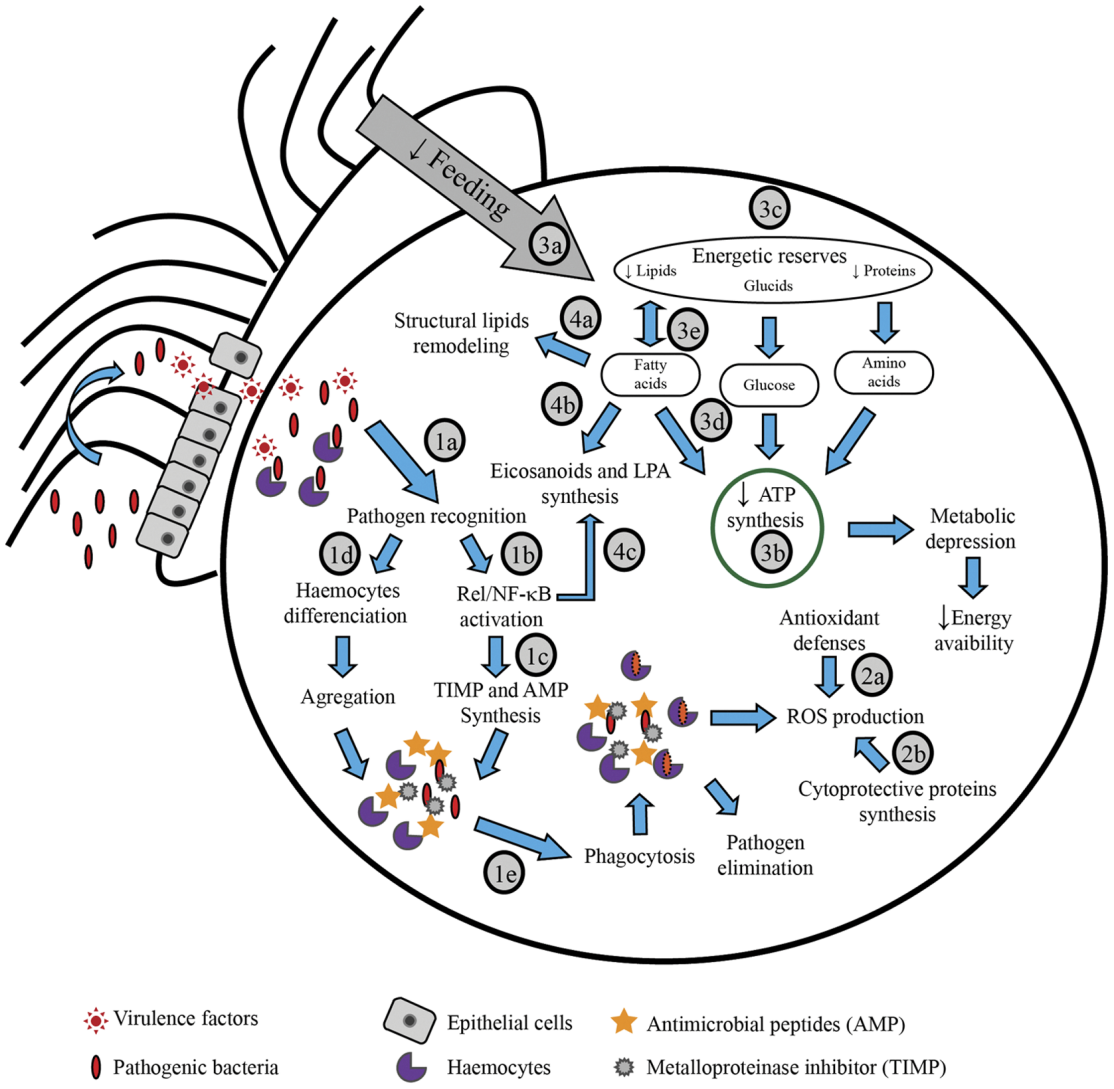
Schematic diagrams of physiological changes induced during bacterial infection in *C. gigas* larvae. Infection impact on larval physiology was investigated through immunity (1), cellular stress (2), energetic metabolism (3) and lipid metabolism (4) using physiological, enzymatic, biochemical and molecular analysis. Results suggest that bacterial infection induce the activation of the immune response (non-self recognition (1a), NF-κB signaling pathway (1b), haematopoiesis (1d), synthesis of inhibitor of metalloproteinase, antimicrobial peptide (1c) and phagocytosis (1e) allowing the destruction of pathogenic bacteria. The production of reactive oxygen species (ROS) during the phagocytosis process was managed by antioxidant defenses (2a) and cytoprotective proteins (2b). Infection affects the feeding activity (3a) which change the energy status of larvae (decline of metabolic rate (3b), energy reserve consumption (3c), β-oxidation activation (3d) and lower lipids storage (3e)). Besides metabolic changes, fatty acid remodeling in polar lipids (4a) is associated to pathogen exposure, as suggested by changes in phosphatidylinositol and lysophosphatidylcholine composition, non-methylene–interrupted fatty acids accumulation, lower content of major C_20_ polyunsaturated fatty acids and activation of desaturases. Finally, infection induces the activation of phospholipase and lipoxygenase (4b) probably through NF-κB regulation (4c) to initiate eicosanoïdes and lysophosphatidic acid (LPA) pathways. See discussion for details.

The main interest and novelty of our study is that we characterized the response of oyster larvae to pathogen infection using a multidisciplinary approach. For the first time, the impact of a bacterial challenge on larval physiology was characterized in a bivalve mollusk at the physiological, enzymatic, biochemical and transcriptional levels. This study has shown the activation of several physiological processes which co-occurred after bacterial infection in oyster larvae. Indeed, we have observed that *V. coralliilyticus* exposure coincided with changes in energy status of larvae, fatty acid remodeling and the activation of antioxidant defenses and immune response. Taken together, all of these results can be gathered in a schematic diagram (Figure 4) which illustrates putative relationships between these physiological processes. This overall view open the way to further studies that will improve our knowledge about the impact of bacterial infection on larval physiology for marine invertebrates and could be extended to other species with other pathogens and at different larval stages. However, although this study allowed to have an overall view of physiological changes associated to bacterial infection, several results requires additional experiments for validation. Specifically, the activation of immune response could be completed by proteomic or other physiological approaches in order to affine knowledge about regulation pathways, especially to highlight the target (including eicosanoids) of the Rel/NF-κB pathway. In the same idea, it could be interesting to develop Dynamic energy budget (DEB) model to investigate precisely the impact of infection on energy metabolism of larvae. Finally, the relatively low mortality rate observed in our experimental challenge was associated with the activation of immune response, antioxidant defenses and cytoprotection processes, suggesting that larvae developed an effective defense strategy to struggle the bacterial infection. Our results contrast with those reported on *C. virginica* larvae during massive mortality event by Genard *et al.* [11], [15], suggesting that physiological changes depend on the infection intensity and resulting mortality, as recently demonstrated in adult *C. gigas* [95], [96] and *C. virginica* [97].

## Supporting Information

File S1 BLAST results and sequencing.
(PDF)

File S2 Response variables: means and statistics.
(PDF)

File S3 RDA biplot illustrating temporal changes in challenged and unchallenged larvae.
(PDF)
