# Supplementary material for: Integrative Study of Physiological Changes Associated with Bacterial Infection in Pacific Oyster Larvae
Source: PLoS One. 2013 May 21;8(5):e64534. doi: 10.1371/journal.pone.0064534 (PMC3660371; doi:10.1371/journal.pone.0064534)
Supplement: File S1 — BLAST results and sequencing. (PDF) [file pone.0064534.s001.pdf]

Table S1.1. BLAST results for retrieved band B1.

Accession	Description	**Max score**	**Total score**	**Query coverage**	**E value**	**Max ident**
EF114162.1	Vibrio coralliilyticus strain WMB27B small subunit ribosomal RNA gene, partial sequence	289	289	100%	6e-75	94%
DQ079633.1	Vibrio coralliilyticus 16S ribosomal RNA gene, partial sequence	289	289	100%	6e-75	94%

Table S1.2. BLAST results for retrieved band B2.

**Accession**	**Description**	**Max score**	**Total score**	**Query coverage**	**E value**	**Max ident**
EF114162.1	Vibrio coralliilyticus strain WMB27B small subunit ribosomal RNA gene, partial sequence	300	300	100%	3e-78	95%
DQ079633.1	Vibrio coralliilyticus 16S ribosomal RNA gene, partial sequence	300	300	100%	3e-78	95%

Table S1.3. BLAST results for *V. coralliilyticus* original strain (HF549288).

**Accession**	**Description**	**Max score**	**Total score**	**Query coverage**	**E value**	**Max ident**
EF114162.1	Vibrio coralliilyticus strain WMB27B small subunit ribosomal RNA gene, partial sequence	333	333	100%	2e-88	100%
DQ079633.1	Vibrio coralliilyticus 16S ribosomal RNA gene, partial sequence	333	333	100%	2e-88	100%

```
               ....|....| ....|....| ....|....| ....|....| ....|....| ....|....|
                       5         15         25         35         45         55
EF114162.1     ---------- ---------- ---------- --------CC AGCCTGATGC AGCCATGCCG
B1             TACGGGAGGC AGCAGTGGGG AATATTGCAC AATTGGGGCC AGCCTGATGC AGCCATGCCG
B2             TACGGGAGGC AGCAGTGGGG AATTTTGCAC AATTGGCGCC AGCCTGATGC AGCAATGCCG
HF549288       ---------- ---------- ---------- ----GGCGCC AGCCTGATGC AGCCATGCCG

               ....|....| ....|....| ....|....| ....|....| ....|....| ....|....|
                      65         75         85         95        105        115
EF114162.1     CGTGTATGAA GGAGGCCTTC GGGTTGTAAG GTCCTTTCAG CAGTGAGGAA GGTGGTAGTG
B1             CGTGTGTGAA GGAGGCCTTC GGGTTGTAAG GCCCTTTCAG CAATGAGGAA CGTTGTACTG
B2             CGTGCATGAA GGAGGCCTTC GGGTTGTAAG CTCCTTTCAG CAGTAAGGAA CGTGGTAGTG
HF549288       CGTGTATGAA GGAGGCCTTC GGGTTGTAAG GTCCTTTCAG CAGTGAGGAA GGTGGTAGTG

               ....|....| ....|....| ....|....| ....|....| ....|....| ....|....|
                     125        135        145        155        165        175
EF114162.1     TTAATAGCAC TATCATTTGA CGTTAGCTGC AGAAGAGGCA CCGGCTAACT CCGTGCCAGC
B1             TTAATAGCAC TAGGATTTGA CGTTAGTTGC AGAAGAGGCA CCGGCTAACT CCGTGCCAGC
B2             TTAATAGCCT TATCATTTGA CGTTAGCTGC AGAAGAGGCA CCGGCTAACT CCGTGCCAGC
HF549288       TTAATAGCAC TATCATTTGA CGTTAGCTGC AGAAGAGGCA CCGGCTAACT CCGTGCCAGC

               ....|....| ....|....| ....|....| ....|....| ....|....| ....|....|
                     185        195        205        215        225        235
EF114162.1     AGCCGCGGTA ATACGGAAGG TGCGAGCGTT AATCGGAATT ACTGGGCGTA AAGCGCATGC
B1             GACCGCGGTA AT-------- ---------- ---------- ---------- ----------
B2             GACCGCGGTA AT-------- ---------- ---------- ---------- ----------
HF549288       AGCCGCGGTA ATACGGAAGG TGCGAGCGTT AATC------ ---------- ----------
```

Figure S1.1. Alignment of *Vibrio corallilyticus* 16s sequences. EF114162.1correspond to the strain with the maximum score after BLAST, B1 and 2 to the sequences of DGGE retrieved bands and HF549288 to the sequence of the original strain used for bacterial challenge.
