# Supplementary material for: Integrative Study of Physiological Changes Associated with Bacterial Infection in Pacific Oyster Larvae
Source: PLoS One. 2013 May 21;8(5):e64534. doi: 10.1371/journal.pone.0064534 (PMC3660371; doi:10.1371/journal.pone.0064534)
Supplement: File S2 — Response variables: means and statistics. (PDF) [file pone.0064534.s002.pdf]

S2. Response variables: means and statistics.

Table S2.1. Summary of means (± STD) and statistics for larval performance and energetic metabolism.

			Mean ± STD					RDA results				Multiple comparisons			
Name	RV	Unity	Ref	24h Chal	24h Unchal	48h Chal	48h Unchal	Time	Challenge	Interaction	All	24h Chal	24h Unchal	48h Chal	48h Unchal
Mortality rate	Mortality	% of dead larvae	0	13.23 ± 0.34	4.56 ± 0.02	16.89 ± 2.07	7.28 ± 1.07	10.53	86.39	0.23	97.14	b	c	a	c
Growth	Shell lenght	µm	133.42	135.71 ± 18.41	134.89 ± 18.18	139.38 ± 18.89	137.41 ± 18.81	16.04	5.18	0.20	21.42	a	a	a	a
Clearence rate	Feeding	n cell larva-1 day-1	5102.04	5298.86 ± 565.15	4912.41 ± 1367.3	1440.49 ± 659.48	4813.07 ± 347.19	35.44	20.17	31.97	87.58	a	a	b	a
ATP synthase	AS6	Relative expression	1	0.86 ± 0.09	0.67 ± 0.32	1.26 ± 0.73	9.60 ± 2.92	35.54	27.18	29.78	92.51	b	b	b	a
Cytochrome C oxidase	CCOi	Relative expression	1	0.83 ± 0.1	0.61 ± 0.12	0.76 ± 0.21	2.72 ± 0.09	34.24	24.84	39.64	98.72	b	b	b	a
Citrate synthase	CS-G	Relative expression	1	0.86 ± 0.41	0.48 ± 0.22	3.44 ± 1.32	4.22 ± 0.18	87.85	0.36	2.99	91.20	b	b	a	a
Enolase	Enolase	Relative expression	1	0.48 ± 0.35	1.19 ± 0.01	0.96 ± 0.28	0.89 ± 0.25	2.34	25.91	38.51	66.75	a	a	a	a
Glutamine synthetase	GS	Relative expression	1	0.58 ± 0.26	0.95 ± 0.53	0.52 ± 0.17	0.54 ± 0.12	17.76	11.84	9.28	38.88	a	a	a	a
NADH oxidoreductase	NADHox	Relative expression	1	1.10 ± 0.19	0.63 ± 0.3	0.89 ± 0.03	2.27 ± 0.67	27.43	10.95	45.96	84.34	b	b	b	a
Carbohydrates	Glucids	ng glucids larva-1	1.68	1.28 ± 0.3	1.44 ± 0.1	1.21 ± 0.09	1.34 ± 0.38	4.38	12.45	0.21	17.05	a	a	a	a
Protein	Protein	ng protein larva-1	39.57	40.9 ± 1.57	37.86 ± 0.33	38.01 ± 0.21	43.43 ± 2.49	7.02	5.55	70.17	82.74	ab	b	b	a
Pyruvate kinase	PK-A	pmol larve-1 min-1	4.14	5.13 ± 0.62	3.22 ± 0.06	6.05 ± 0.41	8.69 ± 0.32	64.31	0.82	32.78	97.92	b	c	b	a
Cytochrome C oxidase	CCO-A	pmol larve-1 min-1	0.29	0.45 ± 0.05	0.49 ± 0.04	0.47 ± 0.03	0.62 ± 0.02	27.50	44.72	13.75	85.96	b	b	b	a
Citrate synthase	CS-A	pmol larve-1 min-1	11.67	9.15 ± 1.93	8.2 ± 0.6	8.69 ± 0.64	9.44 ± 0.36	4.80	0.29	22.60	27.69	a	a	a	a

RDA results correspond to the variance percentage of each response variable (RV) explained by RDA for each explicative variable (time, challenge and interaction of both) considered individually or together (all). Multiple comparisons show the significant differences (letters) between each combination of treatments. Abbreviations: Ref, larvae before bacterial challenge (reference sample); 24 and 48h chal, larvae challenged during 24 or 48h ; 24 and 48h unchal, unchallenged larvae after 24 and 48h.

Table S2.2. Summary of means (± STD) and statistics for neutral lipids.

			Mean ± STD					RDA results				Multiple comparisons			
Name	RV	Unity	Ref	24h Chal	24h Unchal	48h Chal	48h Unchal	Time	Challenge	Interaction	All	24h Chal	24h Unchal	48h Chal	48h Unchal
Alcohols	AL	mass %	1.69	1.13 ± 0.18	1.02 ± 0.32	1.73 ± 0.45	1.36 ± 0.2	44.97	12.08	3.63	60.68	a	a	a	a
Mono-diacylglycerols	DG-MG	mass %	3.09	3.43 ± 0.18	3.76 ± 0.19	2.88 ± 0.24	2.22 ± 0.4	72.44	1.78	16.20	90.41	ab	a	b	b
Free fatty acids	FFA	mass %	4.83	2.53 ± 0.02	3.07 ± 0.15	6.51 ± 0.08	2.16 ± 0.03	19.71	30.43	49.73	99.87	c	b	a	d
Sterols	ST	mass %	23.24	13.32 ± 1.34	13.69 ± 1.83	17.61 ± 0.82	13.87 ± 2.48	27.70	15.67	23.37	66.75	a	a	a	a
Triacylglycerols	TAG	mass %	67.15	79.59 ± 1.69	78.47 ± 2.49	71.28 ± 0.94	80.39 ± 3.12	16.41	25.65	42.12	84.18	a	a	b	a
Sum of neutral lipids	N-Tot	ng lipids larva-1	3.86	4.71 ± 1.1	4.35 ± 0.89	2.09 ± 0.13	4.70 ± 0.23	22.29	21.90	37.86	82.06	a	a	b	a
14:0	N-14_0	mass %	9.11	7.51 ± 0.55	7.65 ± 1.59	7.41 ± 0.19	8.59 ± 0.49	7.29	17.96	11.16	36.41	a	a	a	a
16:0	N-16_0	mass %	11.28	11.36 ± 0.58	11.08 ± 0.02	11.42 ± 0.12	12.02 ± 0.3	36.39	3.62	28.12	68.13	ab	b	a	a
16:1(n-7)	N-16_1(n_7)	mass %	4.84	4.04 ± 0.31	3.99 ± 0.01	3.6 ± 0.1	3.75 ± 0.14	60.58	1.14	5.03	66.76	a	a	a	a
16:2(n-7)	N-16_2(n_7)	mass %	1	0.81 ± 0.03	0.84 ± 0.03	0.89 ± 0.02	0.62 ± 0.07	12.51	31.48	48.11	92.09	a	a	a	b
18:0	N-18_0	mass %	2.26	2.66 ± 0.63	2.15 ± 0.07	1.93 ± 0.81	2.51 ± 0.34	3.77	0.11	32.41	36.29	a	a	a	a
18:1(n-7)	N-18_1(n_7)	mass %	6.96	6.85 ± 0.16	6.77 ± 0.28	6.79 ± 0.01	6.76 ± 0.16	1.91	4.68	1.49	8.08	a	a	a	a
18:1(n-9)	N-18_1(n_9)	mass %	9.53	10.47 ± 0.26	10.82 ± 0.34	10.48 ± 0.27	10.48 ± 0.23	11.39	12.50	12.47	36.36	a	a	a	a
18:2(n-6)	N-18_2(n_6)	mass %	3.79	4.02 ± 0.24	4.02 ± 0.19	4.13 ± 0.06	4.12 ± 0.11	16.36	0.01	0.02	16.38	a	a	a	a
18:3(n-3)	N-18_3(n_3)	mass %	5.56	5.35 ± 0.16	5.49 ± 0.21	5.09 ± 0.06	5.1 ± 0.14	66.22	3.72	2.40	72.34	a	a	a	a
18:4(n-3)	N-18_4(n_3)	mass %	15.97	14.55 ± 0.31	14.84 ± 0.39	12.9 ± 0.19	13.43 ± 0.48	84.29	5.99	0.48	90.76	a	a	b	b
20:1(n-7)	N-20_1(n_7)	mass %	3	3.37 ± 0.09	3.36 ± 0.15	4 ± 0.13	3.58 ± 0.16	59.38	14.62	14.06	88.06	b	b	a	b
20:4(n-3)	N-20_4(n_3)	mass %	1.04	1.18 ± 0.02	1.16 ± 0.03	1.24 ± 0.04	1.13 ± 0.01	1.86	52.98	24.59	79.42	a	ab	a	b
20:4(n-6)	N-AA	mass %	0.93	0.89 ± 0.04	1.17 ± 0.01	1 ± 0.11	0.13 ± 0.01	48.52	33.90	2.19	84.60	b	b	a	ab
20:5(n-3)	N-EPA	mass %	3.76	4.72 ± 0.31	4.90 ± 0.01	5.89 ± 0.05	4.83 ± 0.25	31.47	19.93	40.18	91.57	b	b	a	b
22:5(n-6)	N-DPA	mass %	1.24	1.48 ± 0.09	1.5 ± 0.08	1.65 ± 0.05	1.56 ± 0.03	49.33	5.57	11.39	66.30	a	a	a	a
22:6(n-3)	N-DHA	mass %	10.51	10.82 ± 0.37	11.11 ± 0.37	11.83 ± 0.28	11.15 ± 0.16	37.91	5.10	31.71	74.72	b	ab	a	a
Sum of non-methylene–interrupted fatty acids	N-ΣNMI	mass %	1.72	2.07 ± 0.19	1.8 ± 0.05	2.2 ± 0	2.04 ± 0.27	24.21	34.12	1.86	60.18	a	a	a	a
Sum of saturated fatty acids	N-ΣSFA	mass %	24.54	23.3 ± 1.88	22.47 ± 1.49	22.42 ± 0.87	24.98 ± 0.34	8.76	9.89	37.84	56.49	a	a	a	a
Sum of monounsaturated fatty acids	N-ΣMUFA	mass %	26.54	26.93 ± 0.77	26.81 ± 1.1	27.09 ± 0.48	26.89 ± 0.51	1.16	2.16	0.10	3.42	a	a	a	a
Sum of polyunsaturated fatty acids	N-ΣPUFA	mass %	48.93	49.77 ± 1.12	50.72 ± 0.39	50.50 ± 0.39	48.13 ± 0.17	17.69	10.18	56.09	83.95	ab	a	a	b
Peroxidation index	N-Pind	PI	213.55	219 ± 6	225 ± 3	230 ± 4	217 ± 2	0.40	8.71	66.85	75.97	ab	a	a	b

RDA results correspond to the variance percentage of each response variable (RV) explained by RDA for each explicative variable (time, challenge and interaction of both) considered individually or together (all). Multiple comparisons show the significant differences (letters) between each combination of treatments. Abbreviations: Ref, larvae before bacterial challenge (reference sample); 24 and 48h chal, larvae challenged during 24 or 48h ; 24 and 48h unchal, unchallenged larvae after 24 and 48h.

Table S2.3. Summary of means (± STD) and statistics for polar lipids.

			Mean ± STD					RDA results				Multiple comparisons			
Name	RV	Unity	Ref	24h Chal	24h Unchal	48h Chal	48h Unchal	Time	Challenge	Interaction	All	24h Chal	24h Unchal	48h Chal	48h Unchal
Ceramide aminoethylphosphonate	CAEP	mass %	13.73	11.72 ± 2.43	10.81 ± 0.73	11.61 ± 0.91	9.48 ± 1.16	6.84	30.68	5.00	42.53	a	a	a	a
Cardiolipin	CARDIO	mass %	8.14	5.55 ± 0.74	6.1 ± 0.86	6.17 ± 1.07	5.06 ± 0.48	2.12	3.60	32.12	37.84	a	a	a	a
Lysophosphatidylcholine	LPC	mass %	10.45	4.04 ± 0.98	6.7 ± 0.23	5.31 ± 1.44	10.26 ± 1.12	24.55	60.81	5.54	90.90	b	b	b	a
Phosphatidylcholine	PC	mass %	14.08	24.65 ± 0.84	28.75 ± 5.43	26.64 ± 3.37	29.36 ± 6.71	2.97	20.39	0.84	24.20	a	a	a	a
Phosphatidylserine	PE	mass %	20.63	29.03 ± 2.91	25.93 ± 1.74	28.08 ± 1.13	27.06 ± 2.3	0.06	29.59	7.52	37.17	a	a	a	a
Phosphatidylinositol	PI	mass %	25.07	18.14 ± 0.84	15.06 ± 2.02	17.03 ± 0.87	13.84 ± 1.98	8.52	61.70	0.02	70.24	a	ab	a	b
Phosphatidylserine	PS	mass %	5.19	4.25 ± 1.18	4.26 ± 0.56	3.25 ± 0.49	2.81 ± 0.09	57.93	1.85	2.02	61.80	a	a	a	a
Sum of polar lipids	TLP	ng lipids larva-1	7.3	8.71 ± 0.58	8.56 ± 0.49	10.77 ± 0.46	10.83 ± 1.6	73.51	0.03	0.17	73.71	a	a	a	a
14:0	P-14_0	mass %	2.33	1.84 ± 0.21	1.93 ± 0.09	1.36 ± 0.1	1.78 ± 0.71	20.75	14.23	5.44	40.42	a	a	a	a
16:0	P-16_0	mass %	12.22	13.27 ± 0.58	11.45 ± 1.65	12.16 ± 0.31	10.83 ± 1.64	12.11	39.95	0.98	53.04	a	a	a	a
16:1(n-7)	P-16_1(n_7)	mass %	1.62	1.16 ± 0.03	1.14 ± 0.07	0.9 ± 0.02	0.84 ± 0.08	90.32	1.99	0.66	92.97	a	a	b	b
17:1(n-7)	P-17_1(n_7)	mass %	4.66	4.85 ± 0.17	8.27 ± 1.42	7.74 ± 0	5.92 ± 1.57	0.76	6.44	69.88	77.08	b	a	a	ab
18:0	P-18_0	mass %	3.5	2.44 ± 0.03	2.31 ± 0.01	2.37 ± 0.2	2.36 ± 0.33	0.08	6.12	4.57	10.77	a	a	a	a
18:1(n-7)	P-18_1(n_7)	mass %	5.94	5.57 ± 0.2	5.43 ± 0	5.35 ± 0.09	4.38 ± 0.04	44.88	33.49	18.93	97.30	a	a	a	b
18:1(n-9)	P-18_1(n_9)	mass %	4.81	2.94 ± 0.03	2.46 ± 0.17	2.43 ± 0.06	2.27 ± 0.14	44.67	37.22	8.84	90.73	a	b	b	b
18:2(n-6)	P-18_2(n_6)	mass %	2.05	1.41 ± 0.06	1.24 ± 0.1	1.04 ± 0.13	2.27 ± 0.12	12.32	31.08	54.16	97.56	b	b	b	a
18:3(n-3)	P-18_3(n_3)	mass %	3.2	1.89 ± 0.03	1.9 ± 0.05	1.47 ± 0.02	2.11 ± 0.04	5.04	49.03	44.60	98.67	b	b	c	a
18:4(n-3)	P-18_4(n_3)	mass %	8.2	4.69 ± 0.09	5.25 ± 0.35	4.05 ± 0.05	5.41 ± 0.32	4.75	73.03	12.89	90.67	b	ab	b	a
20:1(n-11)	P-20_1(n_11)	mass %	1.85	1.71 ± 0.17	1.78 ± 0.2	2.12 ± 0.2	1.74 ± 0.05	20.91	14.60	31.31	66.82	a	a	a	a
20:1(n-7)	P-20_1(n_7)	mass %	4.93	4.07 ± 0.11	3.93 ± 0.12	4.22 ± 0.17	3.4 ± 0.11	8.23	55.72	27.78	91.73	a	a	a	b
20:4(n-6)	P-AA	mass %	2.34	2.43 ± 0.09	2.33 ± 0.01	2.12 ± 0.09	3.86 ± 0.12	19.47	35.46	44.23	99.16	b	b	b	a
20:5(n-3)	P-EPA	mass %	6.84	7.8 ± 0.39	7.87 ± 0.03	8.25 ± 0.05	8.83 ± 0.23	64.27	13.64	8.38	86.29	b	b	ab	a
22:2 NMIJ	P-22_2j	mass %	5.21	7.86 ± 0.01	5.91 ± 0.49	8.26 ± 0.25	5.57 ± 0.2	0.02	94.53	2.42	96.97	a	b	a	b
22:5(n-6)	P-DPA	mass %	2.36	2.54 ± 0.17	2.86 ± 0.06	2.18 ± 0.07	3.53 ± 0.05	2.44	69.53	25.92	97.89	c	b	d	a
22:6(n-3)	P-DHA	mass %	18.01	19.64 ± 0.69	19.65 ± 0.01	19.95 ± 0.01	22.97 ± 1.15	37.80	26.23	25.76	89.79	b	b	b	a
Sum of non-methylene–interrupted fatty acids	P-ΣNMI	mass %	7.37	10.91 ± 0.09	8.14 ± 0.51	11.62 ± 0.22	7.53 ± 0.25	0.02	94.89	3.56	98.47	a	b	a	b
Sum of saturated fatty acids	P-ΣSFA	mass %	19.9	19.44 ± 0.97	17.19 ± 1.87	17.5 ± 0.17	16.38 ± 2.98	16.15	24.19	2.71	43.06	a	a	a	a
Sum of monounsaturated fatty acids	P-ΣMUFA	mass %	25.23	21.75 ± 0.30	24.41 ± 1.03	24.28 ± 0.24	19.79 ± 1.53	6.65	5.13	77.47	89.25	b	a	ab	b
Sum of polyunsaturated fatty acids	P-ΣPUFA	mass %	54.87	58.81 ± 0.67	58.40 ± 0.84	58.22 ± 0.07	63.84 ± 1.45	25.14	29.03	38.84	93.01	b	b	b	a
Peroxidation index	P-Pind	PI	273.69	292 ± 7	301 ± 6	293 ± 1	333 ± 12	21.70	49.21	20.40	91.31	b	b	b	a

RDA results correspond to the variance percentage of each response variable (RV) explained by RDA for each explicative variable (time, challenge and interaction of both) considered individually or together (all). Multiple comparisons show the significant differences (letters) between each combination of treatments. Abbreviations: Ref, larvae before bacterial challenge (reference sample); 24 and 48h chal, larvae challenged during 24 or 48h ; 24 and 48h unchal, unchallenged larvae after 24 and 48h.

Table S2.4. Summary of means (± STD) and statistics for lipids related genes.

			Mean ± STD					RDA results				Multiple comparisons			
Name	RV	Unity	Ref	24h Chal	24h Unchal	48h Chal	48h Unchal	Time	Challenge	Interaction	All	24h Chal	24h Unchal	48h Chal	48h Unchal
AA lipoxigenase	AA15LX	Relative expression	1	2.19 ± 0.57	1.37 ± 0.35	8.76 ± 3.08	1.69 ± 0.47	28.07	36.80	23.15	88.02	b	b	a	b
Acyl-CoA dehydrogenase	ADH	Relative expression	1	2.94 ± 0.20	1.99 ± 0.97	5.90 ± 1.93	1.96 ± 0.28	16.84	46.85	17.50	81.18	b	b	a	b
Stearoyl-desaturase 5	Delta5	Relative expression	1	0.58 ± 0.14	0.40 ± 0.36	1.64 ± 0.2	0.47 ± 0.19	28.67	40.34	21.22	90.23	b	b	a	b
Delta 9 desaturase	Delta9	Relative expression	1	0.7 ± 0.46	0.58 ± 0.35	0.79 ± 0.34	1.4 ± 0.86	20.47	6.10	13.66	40.23	a	a	a	a
Endothelial lipase	EDL	Relative expression	1	0.6 ± 0.07	0.29 ± 0.01	0.42 ± 0.04	1.1 ± 1.03	10.81	3.64	26.67	41.12	a	a	a	a
Enoyl-hydratase isomerase	ECH	Relative expression	1	2.89 ± 1.38	0.55 ± 0.22	4.09 ± 0.38	0.67 ± 0.39	4.34	81.73	2.86	88.93	a	b	a	b
Fatty acid desaturase 2	Delta6	Relative expression	1	0.54 ± 0.01	0.15 ± 0.06	2.67 ± 0.85	0.27 ± 0.01	27.55	42.33	22.25	92.13	b	b	a	b
Adipolipin	Lipstor	Relative expression	1	0.47 ± 0.43	0.23 ± 0.12	1.58 ± 0.10	1.43 ± 0.06	89.96	2.62	0.16	92.74	b	b	a	a
Phosphatidylcholine transferase	PCtrans	Relative expression	1	2.89 ± 3.39	1.03 ± 1.07	4.66 ± 1.93	1.89 ± 1.15	10.70	33.21	1.27	45.18	a	a	a	a
Phospholipase delta 1	PLD1	Relative expression	1	1.56 ± 0.92	1.5 ± 0.24	5.4 ± 0.5	1.96 ± 0.67	40.70	27.00	25.22	92.93	b	b	a	b
Phospholipase a2	PLA2	Relative expression	1	0.51 ± 0.46	0.16 ± 0.09	2.17 ± 0.59	0.09 ± 0.04	20.07	46.95	24.00	91.02	b	b	a	b
Phosphatidylserine decarboxylase	Psdecar	Relative expression	1	1.3 ± 0.94	0.59 ± 0.27	2.45 ± 1.93	0.98 ± 0.61	13.42	26.48	3.26	43.16	a	a	a	a
Acyl-CoA synthetase	ACS	Relative expression	1	2.25 ± 0.65	0.84 ± 0.01	4.11 ± 0.03	2.56 ± 0.53	55.71	38.04	0.08	93.82	b	c	a	b

RDA results correspond to the variance percentage of each response variable (RV) explained by RDA for each explicative variable (time, challenge and interaction of both) considered individually or together (all). Multiple comparisons show the significant differences (letters) between each combination of treatments. Abbreviations: Ref, larvae before bacterial challenge (reference sample); 24 and 48h chal, larvae challenged during 24 or 48h ; 24 and 48h unchal, unchallenged larvae after 24 and 48h.

Table S2.5. Summary of means (± STD) and statistics for parameters associated to immunity and cellular stress.

			Mean ± STD					RDA results				Multiple comparisons			
Name	RV	Unity	Ref	24h Chal	24h Unchal	48h Chal	48h Unchal	Time	Challenge	Interaction	All	24h Chal	24h Unchal	48h Chal	48h Unchal
a-agglutinin	AGL	Relative expression	1	0.77 ± 0.19	0.33 ± 0.35	4.25 ± 0.69	0.29 ± 0.27	26.39	42.96	27.54	96.89	b	b	a	b
Annexin 6	ANX6	Relative expression	1	0.76 ± 0.27	0.47 ± 0.32	1.95 ± 0.4	1.25 ± 0.31	65.60	16.81	2.92	85.33	b	b	a	ab
Cg-defensin 2	Defh2	Relative expression	1	0.60 ± 0.26	0.14 ± 0.04	1.81 ± 0.49	0.30 ± 0.03	24.39	50.55	14.58	89.52	b	b	a	b
Cg-DRAC3	DRAC3	Relative expression	1	1.17 ± 0.42	2.73 ± 0.47	1.10 ± 0.03	0.59 ± 0.2	43.98	9.87	38.43	92.28	b	a	b	b
ECSIT	ECSIT	Relative expression	1	3.11 ± 1.32	1.71 ± 1.64	1.46 ± 1.46	2.49 ± 1.92	2.76	0.51	21.53	24.79	a	a	a	a
Galectin 8	GAL8	Relative expression	1	1.11 ± 0.61	1.07 ± 0.06	0.65 ± 0.48	0.03 ± 0.01	53.28	10.14	8.11	71.53	a	a	ab	b
LPS binding/bactericidal-permeability-increasing protein	LBP/BPI	Relative expression	1	8.28 ± 2.34	1.50 ± 0.34	8.86 ± 4.31	2.14 ± 0.9	0.63	78.01	0.00	78.65	a	b	a	ab
Mitogen activated protein kinase kinase 1	MAPK	Relative expression	1	0.8 ± 0.05	2.81 ± 3.51	2.81 ± 3.25	1.78 ± 1.55	1.53	1.56	15.06	18.15	a	a	a	a
Myeloid differentiation primary response (88)	MYD88	Relative expression	1	11.33 ± 1.09	1.58 ± 1.39	2.01 ± 0.17	0.46 ± 0.39	35.13	41.14	21.61	97.89	a	b	b	b
Cg-REL	REL	Relative expression	1	0.96 ± 0.59	0.63 ± 0.21	3.61 ± 0.61	1.81 ± 0.31	63.55	19.56	9.45	92.56	b	b	a	b
Cg-TAL	TAL	Relative expression	1	0.37 ± 0.28	0.18 ± 0.05	1.81 ± 0.73	0.37 ± 0.06	32.87	32.66	19.26	84.79	b	b	a	b
Tissue inhibitor metalloproteinase	TIMP	Relative expression	1	1.55 ± 0.39	0.35 ± 0.11	6.03 ± 1.58	1.68 ± 0.71	41.75	38.20	12.19	92.14	b	b	a	b
TNF receptor associated factor	TRAF	Relative expression	1	0.45 ± 0.05	0.52 ± 0.09	3.52 ± 0.59	0.95 ± 0.85	43.39	22.12	24.41	89.92	b	b	a	b
c-type lectin-1	cLEC	Relative expression	1	3.44 ± 0.52	0,01 ± 0,01	0.09 ± 0.03	0.01 ± 0,01	31.68	35.03	31.77	98.48	a	b	b	b
Catalase	CAT-G	Relative expression	1	3.04 ± 0.40	0.65 ± 0.07	7.45 ± 0.70	1.72 ± 1.06	26.83	59.05	10.06	95.95	b	b	a	b
Superoxide dismutase extracellular	ecSOD	Relative expression	1	0.3 ± 0.42	0.14 ± 0.18	0.36 ± 0.5	0.49 ± 0.18	14.50	0.08	6.81	21.40	a	a	a	a
Glutathione peroxidase 3	GPX3	Relative expression	1	0.66 ± 0.32	0.73 ± 0.36	1.55 ± 0.92	0.99 ± 0	32.13	5.88	9.57	47.59	a	a	a	a
Glutathione peroxidase 5	GPX5	Relative expression	1	1.59 ± 0.08	0.83 ± 0.53	2.7 ± 0.21	1.74 ± 0.07	52.31	38.44	0.52	91.27	b	b	a	b
Glutathione reductase	GR-G	Relative expression	1	0.57 ± 0.24	0.13 ± 0.02	2.19 ± 0.66	0.15 ± 0.17	21.73	49.23	20.61	91.57	b	b	a	b
Heat Shock protein 70	HSP70	Relative expression	1	1.24 ± 0.95	1.02 ± 0.46	5.79 ± 1.50	0.83 ± 0.06	25.28	35.80	29.92	91.00	b	b	a	b
Metallothionein	Metallo	Relative expression	1	0.64 ± 0.31	0.61 ± 0.54	0.93 ± 0.16	1.3 ± 0.01	46.21	5.44	7.87	59.53	a	a	a	a
Peroxiredoxin 4	PRDX4	Relative expression	1	0.45 ± 0.3	0.36 ± 0.26	2.79 ± 0.14	1.59 ± 0.25	79.04	10.33	7.73	97.09	c	c	a	b
Peroxiredoxin 5	PRDX5	Relative expression	1	0.79 ± 0.06	0.86 ± 0.89	2.61 ± 0.19	0.82 ± 0.10	27.95	26.47	30.72	85.14	b	b	a	b
Peroxiredoxin 6	PRDX6	Relative expression	1	0.56 ± 0.06	0.42 ± 0.32	1.55 ± 0.16	0.94 ± 0.14	67.91	16.63	6.49	91.03	b	b	a	b
Pernin	PRN	Relative expression	1	0.65 ± 0.1	0.09 ± 0.04	1.64 ± 0.30	0.67 ± 0.21	46.35	44.91	3.17	94.43	b	c	a	b
Superoxide dismutase	SOD-G	Relative expression	1	0.64 ± 0.52	0.19 ± 0.13	4.18 ± 0.86	0.47 ± 0.38	32.36	38.69	23.75	94.80	b	b	a	b
Lipids peroxidation	TBARS	pmol larva-1	0.03	0.03 ± 0	0.02 ± 0	0.04 ± 0	0.04 ± 0	78.89	7.80	1.14	87.83	b	b	a	ab
Glutathione peroxidase	GPX-A	pmol larve-1 min-1	1.89	2.14 ± 0.33	2.44 ± 0.01	3.97 ± 0.37	4.28 ± 0.22	93.33	2.58	0.00	95.91	b	b	a	a
Glutathione reductase	GR-A	pmol larve-1 min-1	1.79	2.37 ± 0.46	1.99 ± 0.33	2.61 ± 0.25	3.41 ± 0.02	53.96	3.42	27.37	84.75	b	b	ab	a
Superoxide dismutase	SOD-A	µU larve-1 min-1	1193.29	1947 ± 328	1549 ± 21	2212 ± 133	1146 ± 378	0.61	68.14	14.17	82.92	a	ab	a	b
Catalase	CAT-A	pmol larve-1 min-1	60.92	102.51 ± 5.19	86.86 ± 5.89	123.28 ± 0.55	93.72 ± 3.15	24.27	64.99	6.16	95.43	b	b	a	b

RDA results correspond to the variance percentage of each response variable (RV) explained by RDA for each explicative variable (time, challenge and interaction of both) considered individually or together (all). Multiple comparisons show the significant differences (letters) between each combination of treatments. Abbreviations: Ref, larvae before bacterial challenge (reference sample); 24 and 48h chal, larvae challenged during 24 or 48h ; 24 and 48h unchal, unchallenged larvae after 24 and 48h.
