# Supplementary material for: Integrative Study of Physiological Changes Associated with Bacterial Infection in Pacific Oyster Larvae
Source: PLoS One. 2013 May 21;8(5):e64534. doi: 10.1371/journal.pone.0064534 (PMC3660371; doi:10.1371/journal.pone.0064534)
Supplement: File S3 — RDA biplot illustrating temporal changes in challenged and unchallenged larvae. (PDF) [file pone.0064534.s003.pdf]

S3. RDA biplot illustrating temporal changes in challenged and unchallenged larvae.

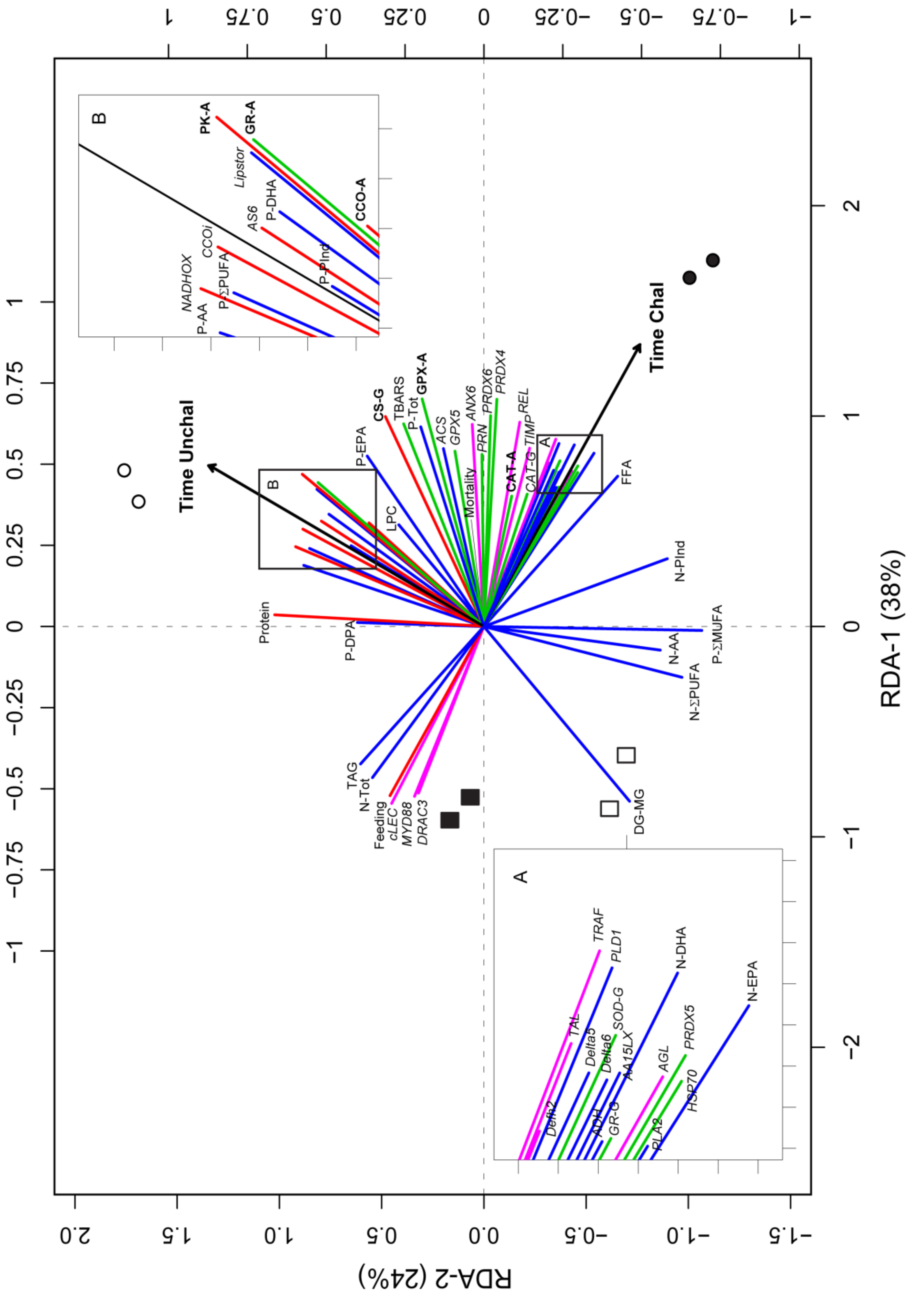

Figure S3.1. Ordination biplot representing the significant ($p<0.05$) experimental time effect on challenged (Time Chal) and unchallenged (Time Unchal) larvae as explanatory variables (arrows) on selected response variables (lines) related to energy metabolism (red), lipids (blue), cellular stress (green), and immunity (pink). Response variables related to larval performance and biochemical analysis were formatted in normal text, to enzymatic analysis in bold and to molecular analysis in italic. Black symbols: challenged larvae, white symbols: unchallenged larvae, squares: 24h of exposure, circles: 48h of exposure. Response variables abbreviations: -A, enzymatic activity ; -G, gene expression ; N-, neutral lipids; P-, polar lipids; S, sum of; cLEC, c-type lectin-1; AGL, a-agglutinin attachment subunit; MYD88, Myeloid differentiation primary response (88); TRAF, TNF receptor associated factor; REL, REL protein; DRAC3, drosophila rho GTPase 3; TAL, hematopoietic transcription factors; TIMP, tissue inhibitor metalloproteinase; Defh2, defensine 2; ANX6, anexin 6; TBARS, lipids peroxidation, GR, glutathione reductase; SOD, superoxide dismutase; CAT, catalase; GPX(5), glutathione peroxidase (family 5); PRDX4-5-6, peroxiredoxine 4, 5 and 6; PRN, Pernin; HSP70, Heat shock protein 70; NADHOX, Mitochondrial nadh:ubiquinone oxidoreductase; CCOi, Cytochrome c oxidase subunit i; AS6, ATP synthase f0 subunit 6; CS, citrate synthase, PK, pyruvate kinase; CCO, cytochrome c oxidase; ADH, Acyl-CoA dehydrogenase; ECH, Enoyl-hydratase isomerase family protein; ACS, Acyl-CoA synthetase; Lipstor, Adipophilin; Delta5, Stearoyl-desaturase 5; Delta6, Fatty acid desaturase 2; PLA2, Phospholipase a2 receptor 1; PLD1, Phospholipase delta 1; AA15LX, Arachidonate 15-lipoxygenase; TOT, total lipids; DG-MG, mono-diglycerols; LPC, lysophosphatidylcholine; TAG, triacylglycerols; FFA, free fatty acids; AA, arachidonic acid; EPA, eicosapentaenoic acid; DPA, Docosapentaenoic acid; DHA, Docosahexaenoic acid; MUFA, monounsaturated fatty acids; PUFA, polyunsaturated fatty acids; NMI, non-methylene–interrupted fatty acids; Pind, peroxidation index; Protein, total proteins content; Feeding, feeding activity; Mortality, mortality rate.
